# Intercepting Photogenerated Aminyl Radicals at Metal‐Halide Perovskite Microcrystals to Forge C─N Bonds With Non‐Preactivated Substrates

**DOI:** 10.1002/smsc.70306

**Published:** 2026-05-18

**Authors:** Daniele Conelli, Nicola Margiotta, Gian Paolo Suranna, Roberto Grisorio

**Affiliations:** ^1^ Dipartimento di Ingegneria Civile Ambientale del Territorio Edile e di Chimica (DICATECh) Politecnico di Bari Bari Italy; ^2^ Dipartimento di Chimica Università degli Studi di Bari “Aldo Moro” Bari Italy; ^3^ CNR‐NANOTEC – Institute of Nanotechnology Lecce Italy

**Keywords:** aminium radical cation, aminyl radical, cross‐dehydrogenative coupling, metal‐halide perovskite, photocatalysis

## Abstract

Visible‐light‐driven C─N bond formation from non‐preactivated partners remains a central challenge in oxidative synthesis, particularly when selective control over N‐centered radical intermediates is required. Here, lead‐halide perovskite microcrystals are shown to mediate cross‐dehydrogenative C─N coupling of aromatic heterocyclic amines in air at room temperature by controlling complementary radical species at semiconductor interfaces. Phenoxazine undergoes efficient oxidative dimerization, whereas closely related scaffolds such as phenothiazine and 9,9‐dimethyl‐9,10‐dihydroacridine display markedly different reactivity profiles despite comparable oxidation potentials. Combined electrochemical, spectroscopic, and radical‐trapping experiments reveal that efficient C─N bond formation correlates with the concurrent generation of radical cation and neutral aminyl species at the catalyst–substrate interface. UV–vis monitoring under controlled atmospheres establishes the necessity of oxygen for sustaining complementary radical populations, while spin‐unrestricted DFT calculations identify substrate‐dependent spin localization, as well as distinct N─H activation barriers, as key factors of coupling efficiency. Interception of the photogenerated aminyl radicals enables hetero‐cross‐dehydrogenative coupling with naphthol and naphthylamine derivatives. These findings delineate mechanistic principles governing C─N bond formation at semiconductor surfaces and position metal‐halide perovskites as tunable and recyclable platforms for N‐centered radical chemistry under mild conditions.

## Graphical abstract

Visible‐light‐excited CsPbBr_3_ perovskite microcrystals enable selective C─N bond formation from non‐preactivated aromatic amines via controlled generation of complementary aminyl and aminium radicals at the catalyst surface. Radical interplay governs reactivity, allowing efficient homo‐ and hetero‐cross‐dehydrogenative coupling under mild aerobic conditions with recyclable semiconductor photocatalysts.

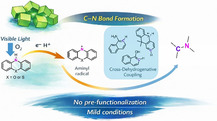

## 1 Introduction

Developing direct and selective methods to forge aryl C─N bonds remains a central objective in synthesis, given their prevalence in pharmaceuticals, agrochemicals, and functional materials [1, 2]. Condensed aromatic heterocycles (e.g., phenoxazine, phenothiazine) exhibit rich and tunable reactivity and often appear as their N‐aryl derivatives. In particular, N‐aryl phenothiazines and phenoxazines are prominent motifs in photocatalysts [3, 4], hole‐transport materials for perovskite solar cells [5, 6], and thermally activated delayed fluorescence (TADF) emitters [7, 8]. Phenothiazine scaffolds are also widely represented in medicinal chemistry, from first‐generation antipsychotics and antihistamines to anthelmintics [9]. Traditionally, these architectures are assembled through transition‐metal‐catalyzed cross‐couplings (Buchwald–Hartwig [10], Ullmann [11], and Chan–Lam [12]), which offer high site selectivity but typically require prefunctionalized partners (aryl halides or arylboron reagents). Undirected aromatic amination via C(*sp* ^2^)─H/N─H cross‐dehydrogenative coupling (CDC) obviates arene prefunctionalization, enabling a rapid evolution of molecular complexity in an atom‐economical step [13, 14]. Beyond the intrinsic challenge of C─H regioselectivity [15, 16], a key limitation of CDC is that the amine partner often must be preactivated or preoxidized at the N─H bond to achieve reactivity [17].

Within this landscape, N‐centered radicals derived from aromatic heterocycles are attractive intermediates for oxidative C─N bond formation. Dehydrogenative phenothiazination (and the phenoxazine analog) has become a benchmark transformation for evaluating feasibility and scope [18]. Under metal‐ and catalyst‐free conditions, the reaction proceeds through a radical pathway at high temperature (150°C), with cumene and molecular oxygen acting in the oxidation manifold [19]. To enable milder conditions and broaden substrate scope while improving yields, subsequent studies introduced copper catalysts [20], organic photocatalysts [21], stoichiometric oxidants (K_2_S_2_O_8_ [22], NO_2_BF_4_ [23], KIO_4_ [24]), and electrochemical protocols with impressive performance across diverse substrates (including aryl amines) [25, 26].

In a representative mechanism, an amine can be converted either to an aminium radical cation via hole transfer in a photo‐oxidative process or to an aminyl radical via a subsequent proton transfer. The resulting N‐centered radical undergoes homolytic aromatic substitution (HAS) on an electron‐rich arene to afford a cyclohexadienyl radical, which rearomatizes through sequential oxidation and deprotonation, delivering a net dehydrogenative C─N coupling [22]. Alternatively, oxidation of an electron‐rich arene to its radical cation, captured by the amine and followed by deprotonation, likewise forges the C─N bond [23], establishing the conventional electrophilic aromatic substitution (EAS) pathway. In both processes, regioselectivity can be tuned by the arene scaffold, the acid–base environment, and the redox window of the (photo)catalyst.

Despite their utility, many oxidative and photochemical syntheses of N‐aryl phenothiazines (and phenoxazines) rely on excess oxidants and on homogeneous (organo)catalysts that are difficult to recover and recycle [27]. By contrast, electrochemical protocols often require supporting electrolytes used in a sacrificial manner, which can compromise overall sustainability [25].

Inorganic lead‐halide perovskites (most notably CsPbBr_3_) have emerged as efficient photocatalysts under visible light, owing to their intermediate band gap (≈2.3 eV), high absorptivity, and relatively long charge‐carrier lifetimes [28, 29]. Favorable redox potentials (valence band maximum up to +1.5 V vs. NHE depending on morphology of the perovskite semiconductor) enable hole transfer to aromatic heterocyclic amines, generally endowed with relatively low oxidation potentials, as well as reduction of molecular oxygen to superoxide. In model systems, CsPbBr_3_ photocatalysts can promote aerobic amine oxidations and related photoredox transformations through effective catalyst–substrate interfaces, highlighting their potential for sustainable and recyclable catalysis [30].

In this study, we demonstrate that metal‐halide perovskite microcrystals enable controlled generation of complementary aminyl and aminium radical species at the catalyst surface, providing a platform for selective cross‐dehydrogenative C─N bond formation from non‐preactivated partners. CsPbBr_3_ powder acts as heterogeneous and recyclable photocatalyst using phenoxazine (POZ) and phenothiazine (PTZ) as N‐containing species to forge the C─N bond under air and blue‐light irradiation at room temperature, albeit with distinct kinetics, involving the corresponding aminyl radicals as the reactive intermediates. These findings emphasize the ability of perovskite‐based photocatalysts to mediate selective C─N bond formation under mild conditions and position CsPbBr_3_ materials as an innovative platform for photoredox C─N coupling from non‐preactivated precursors.

## 2 Results and Discussion

### 2.1 Reaction Optimization

To probe whether perovskite photocatalysts could generate N‐centered radical intermediates from aromatic heterocycles, phenoxazine (POZ) was selected as a model substrate. Under blue‐light irradiation in the presence of CsPbBr_3_ microcrystals, selective formation of the biphenoxazine dimer was observed, demonstrating that perovskite photoexcitation enables oxidative C─N bond formation. Key parameters influencing the reaction outcome are summarized in Table 1. Changing the solvent had a pronounced effect on both conversion and yield of the reaction (entries 1–7 of Table 1). In general, polar aprotic solvents outperform apolar ones, and acetonitrile (MeCN) afforded the highest GC yield (40.1%, entry 7 of Table 1, Figure S1). This trend suggests that solvent polarity stabilizes key charged and/or radical intermediates and favors productive pathways over unselective background processes. Notably, switching from blue to green LEDs maintained high conversion (94.6%) but substantially lowered the yield (16.7%, entry 8 of Table 1), indicating that efficient product formation is wavelength‐dependent and that green light predominantly promotes collateral processes, likely due to less efficient photoinduced charge generation or interfacial charge transfer.

**TABLE 1:** Optimization of reaction conditions for the visible‐light photocatalytic transformation.^a^

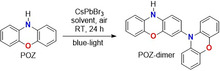				
Entry	Solvent	Variation	Conversion, %	Yield, %
1	DCE	–	73.7	13.6
2	Toluene	–	77.3	13.2
3	EtOAc	–	97.2	9.8
4	CPME	–	85.2	24.2
5	2‐MeTHF	–	85.8	14.3
6	Cyclohexane	–	76.9	9.0
7	MeCN	–	89.0	40.1 (35.1)
8	MeCN	Green light	94.6	16.7
9	MeCN	No cat.	–	NR
10	MeCN	No light	66.1^b^	NR
11	MeCN	Cs_3_Bi_2_Br_9_	65.1	6.1
12	MeCN	Cs_2_AgBiBr_6_	–	NR
13	MeCN	N_2_	64.4	NR
14	MeCN	TEMPO	72.0	NR
15	MeCN	BQ	89.8	32.2

a Reactions were performed under visible‐light irradiation using the indicated solvent and conditions. Conversion and yield were determined by GC analysis using biphenyl as an internal standard; isolated yields are reported in parentheses where applicable.

b Determined to evaluate the substrate adsorption. Solvent abbreviations: 2‐MeTHF, 2‐methyltetrahydrofuran; BQ, 1,4‐benzoquinone; CPME, cyclopentyl methyl ether; DCE, 1,2‐dichloroethane; EtOAc, ethyl acetate; MeCN, acetonitrile; NR, no reaction; TEMPO, 2,2,6,6‐tetramethylpiperidine‐1‐oxyl.

Omitting either the photocatalyst or light suppresses product formation (entries 9 and 10 of Table 1, respectively), no product evolution was observed, corroborating the photocatalytic nature of the transformation and the indispensable coexistence of both the catalyst and light.

The chemical structure of the POZ‐dimer product (isolated in 35.1% yield under the conditions of entry 7 of Table 1) was scrutinized by 1D and 2D multinuclear NMR spectroscopy (Figures S2‐4), identifying in 10H‐3,10'‐biphenoxazine (POZ‐dimer) the target compound. Therefore, the proposed synthetic protocol selectively builds a C─N bond at C3 position of POZ scaffold in the CDC regime imposed by the photocatalyst.

This reactivity was found to be intrinsic to the lead‐based perovskite photocatalyst, as the corresponding bismuth‐based analogs (Cs_3_Bi_2_Br_9_ and Cs_2_AgBiBr_6_) were essentially inactive under identical reaction conditions (entries 11 and 12 of Table 1). The marked difference in performance points to a decisive role of the catalyst surface in determining the fate of photogenerated intermediates, rather than the semiconductor electronic structure governing the initial charge‐transfer event.

**SCHEME 1:**
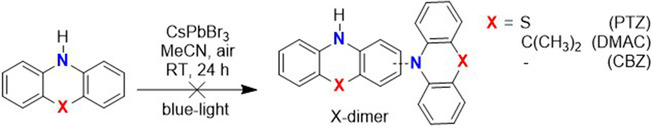
CsPbBr_3_‐photocatalyzed C─N bond formation with structurally analogous substrates.

To probe the generality of this transformation and to assess whether dimerization represented a broader feature of aromatic heterocyclic amines, structurally related scaffolds were examined (Scheme 1). Replacement of POZ with phenothiazine (PTZ) or 9,9‐dimethyl‐9,10‐dihydroacridine (DMAC) led only to trace formation of the corresponding dimer detected by GC–MS analysis. Carbazole (CBZ), despite its structural resemblance, remained completely unreactive. The pronounced divergence among these closely related substrates raised fundamental mechanistic questions: which reactive species were generated upon photoexcitation, how their electronic structures differed across the series, and why only POZ satisfied the requirements for productive radical coupling. Addressing these questions became essential for understanding the origin of selectivity and for establishing design principles for perovskite‐driven C─N bond formation. Accordingly, a comprehensive mechanistic investigation combining spectroscopic, electrochemical, and theoretical analyses was undertaken.

### 2.2 Mechanistic Insights: Experimental Evidence

We proceeded to gain insights into the mechanistic aspects of the synthetic method by first ascertaining the heterogeneous catalytic pathway. It is well known in fact that the relatively low stability of perovskite materials in polar media can determine the diffusion of their constituents in solution [31]. Therefore, we first verified the heterogeneous nature of the proposed photocatalysis by filtering the reaction mixture (upon 1 h stirring in the dark) to remove the solid material and monitoring subsequent reactivity. The filtered solution showed no product evolution, demonstrating that the catalytic activity resides in the solid phase and that any homogeneous contribution from leached species is negligible. We also verified the eventual presence of leached species (halo‐plumbates) under the reaction conditions by UV–vis monitoring. Effectively, a catalyst dispersion in MeCN exhibits absorption features attributable to soluble PbBr_6_^4−^ species [32], indicating some leaching of the solid catalyst under the reaction conditions (Figure S5). However, the concentration of soluble bromo‐plumbate species remains unchanged under irradiation and stirring in the absence of substrate (Figure S5), suggesting that they are in dynamic equilibrium with the surface, without thus perturbing the photocatalyst stoichiometry or exposure of its active catalytic sites during reaction.

Having ascertained the heterogeneous character of the reaction, we next examined catalyst/substrate interactions by determining that, in the absence of photoexcitation, substantial apparent substrate conversion was nonetheless observed (66.1%, entry 10 of Table 1). We attribute this result to strong adsorption of POZ on the photocatalyst surface, as confirmed by diffuse reflectance spectroscopy (DRS) analysis of the catalyst recovered after the reaction carried out in dark (Figure S6). We can therefore assume that substrate adsorption is a key step in its activation by the photoexcited catalyst and that, in the absence of a productive catalytic cycle, the substrate remains trapped on the catalyst surface. Hence, it can be inferred that adsorption strongly influences also the conversion data reported in Table 1, as well as plausible degradation pathways of reactive intermediates under aerobic conditions [29, 33]. Performing the reaction under N_2_ likewise inhibited the product formation (entry 13 of Table 1), confirming the essential role of oxygen as an electron acceptor of the photogenerated charges, thus closing the catalytic cycle.

To probe radical involvement and the possible contribution of superoxide species (developing under aerobic conditions), we examined the effect of proper scavengers (entries 14 and 15 of Table 1). While TEMPO completely suppressed product formation, strongly hinting a radical pathway supporting the transformation, 1,4‐benzoquinone, a superoxide scavenger, moderately decreased the yield (32.2%), suggesting that superoxide plays a minor, but not negligible, role in the overall mechanism.

**FIGURE 1:**
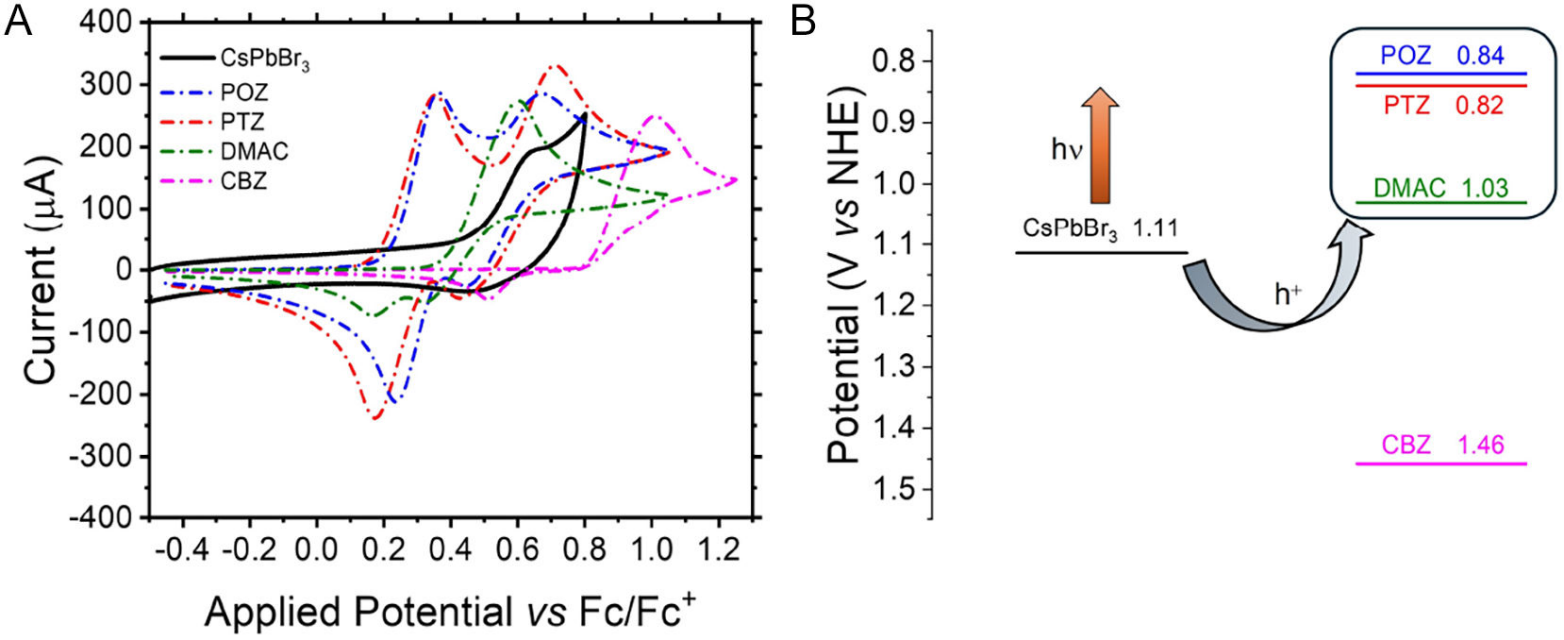
(A) Cyclic voltammograms of CsPbBr_3_, phenoxazine (POZ), phenothiazine (PTZ), carbazole (CBZ), and 9,9‐dimethyl‐9,10‐dihydroacridine (DMAC) recorded in acetonitrile containing 0.1 M n‐Bu_4_NPF_6_. Potentials are referenced to the Fc/Fc^+^ couple. (B) Oxidation onset potentials converted to the NHE scale using the equation: E(NHE) = E(Fc/Fc^+^) + 0.63 V, schematizing the feasibility of the photoinduced hole transfer process with the selected substrates.

To propose a plausible reaction mechanism in line with the collected mechanistic evidence, we are therefore forced to consider a pathway involving radical–radical coupling between photogenerated species of the substrate. As a first step of the reaction mechanism construction, we evaluated the thermodynamic feasibility of the initial charge‐transfer event typical of photocatalytic transformations by comparing the valence‐band potential of the semiconductor (CsPbBr_3_) with the redox properties of the investigated substrates. To this end, cyclic voltammetry (CV) measurements were performed under identical conditions to determine the oxidation potentials of the actors involved in the reaction. As shown in Figure 1A, both POZ and PTZ exhibited closely similar oxidation onset potentials (0.84 and 0.82 V vs. NHE, respectively) and substantially lower with respect to that of the photocatalyst (1.11 V vs. NHE), indicating that initial oxidative activation (hole transfer) by photoexcited CsPbBr_3_ is thermodynamically feasible for both substrates (Figure 1B). The same process is also possible for DMAC, although its oxidation onset potential was found to be higher (1.03 V vs. NHE) in comparison to the other two aromatic heterocycle amines. For these three substrates, indeed, irradiation caused rapid formation of a strongly colored reaction mixture, which can be explained with the accumulation of potentially reactive (aminyl or aminium) intermediates [34].

Conversely, the oxidation onset of CBZ (properly chosen as a diagnostic control substrate) occurs at significantly more positive potential (1.46 V vs. NHE) relative to the operative oxidative window of CsPbBr_3_, placing it beyond the thermodynamically accessible regime for hole transfer. In full agreement with this energetic mismatch, no reaction was observed experimentally, and the reaction mixture remained colorless throughout irradiation time, indicating no formation of reactive intermediates under the applied conditions.

Given the oxidation potentials of PTZ, POZ and DMAC, generation of the same reactive intermediates from photoexcited CsPbBr_3_ would therefore be expected. However, the question remains unresolved as to why PTZ and DMAC, despite satisfying the thermodynamic requirement, are not reactive under these conditions and the answer may include deactivation or degradation of photogenerated intermediates.

**FIGURE 2:**
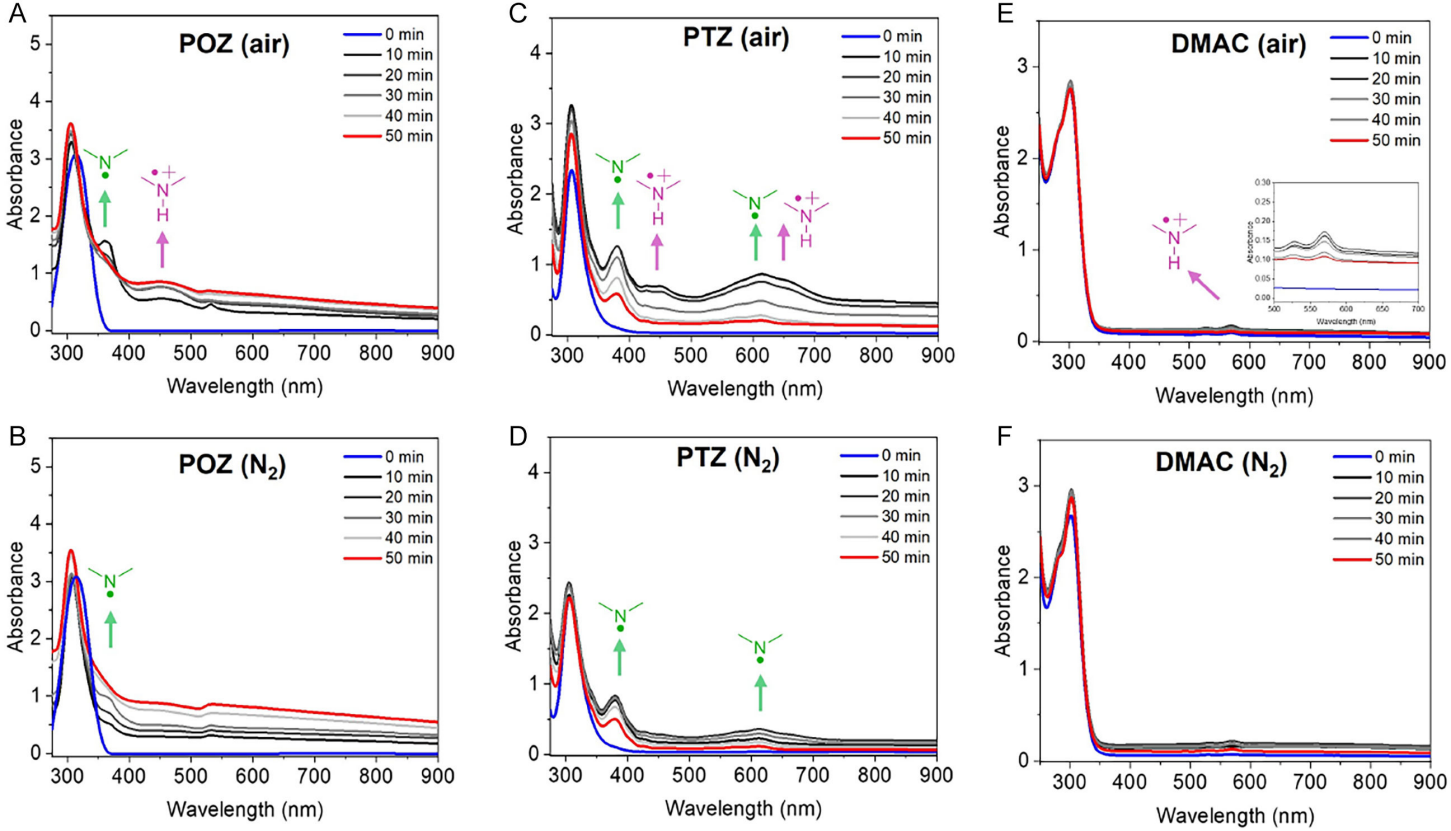
Time‐dependent UV–vis absorption spectra of the reaction mixtures including POZ or PTZ under different atmospheres: (A) POZ in air, (B) POZ under N_2_, (C) PTZ in air, and (D) PTZ under N_2_, (E) DMAC in air, and (F) DMAC under N_2_. Spectra were collected at the indicated times (0–50; 10 min increments). The arrows highlight the behavior of the intensity of the diagnostic bands assigned to the aminium and aminyl radical species.

To elucidate the nature and temporal evolution of photogenerated reactive intermediates, UV–vis absorption spectra of the reaction mixture were recorded under air and under nitrogen at different irradiation time (Figure 2). The comparison between these atmospheres proved diagnostically powerful. Under anaerobic conditions, oxygen was excluded as an external electron acceptor, thereby constraining the photochemical pathway to processes sustained solely by charge separation within the catalyst‐substrate assembly. In this regime, photogenerated electrons could be consumed only through proton‐coupled pathways, while rapid charge recombination limited the steady‐state concentration of radical cation species. As a consequence, the formation of persistent aminium intermediates was intrinsically disfavored in the absence of oxygen, and the detectable transient species were expected to arise predominantly from aminyl radicals generated through photoinduced proton/electron transfer. These N‐centered radicals possess characteristic absorption signatures [35], enabling their direct spectroscopic identification. Thus, monitoring the spectral evolution under inert atmosphere allowed selective interrogation of the aminyl pathway, whereas experiments conducted under air reflected the simultaneous generation and interplay of complementary radical cation and neutral radical intermediates.

In the case of POZ, irradiation of the reaction mixture (with an increased catalyst/substrate ratio to accelerate reaction kinetics, as detailed in Supporting Information) in air produced the initial appearance of broad bands centered at ca. 380 and 450 nm (Figure 2A), which can be assigned to the aminyl (POZ–H^•^, 380 nm) and aminium (POZ^•+^, 450 nm) species, in agreement with the corresponding simulated UV–vis spectra (Figures S7 and S8) [36]. Both intermediates are generated when oxygen is available as a terminal electron acceptor and the absence of their accumulation over time is compatible with the (experimentally observed) product formation consuming them. Parallelly, we established the optical behavior of the POZ‐dimer species under the same photoredox conditions, confirming that the residual absorption bands exhibited by the reaction mixture after 50 min are compatible with the coupling product (Figure S9). Under a nitrogen atmosphere, where no product formation was observed, similar absorption features appeared at ca. 380 nm with lower relative intensity (Figure 2B). This band gradually increased over the first 30 min and then disappeared probably due to decomposition of the aminyl intermediate in the absence of product evolution. Notably, the initial accumulation of POZ–H^•^ is evident in the absence of a reactive partner (aminium ion). These observations support the conclusion that C─N bond formation proceeds efficiently only when complementary radical partners are generated concurrently.

The behavior of PTZ differed sharply (Figure 2C,D) because, under air, absorption features assigned to PTZ^•+^ and PTZ–H^•^ appear only transiently at short irradiation times and then decay, with the spectrum reverting toward that of the neutral substrate, while no dimer product was experimentally observed. This transient profile implies that PTZ‐derived radicals are rapidly consumed in off‐pathway deactivation or degradation processes. As supported by comparison with the simulated UV–vis spectra of PTZ–H^•^ and PTZ^•+^ (Figures S10 and S11), only PTZ–H^•^ formed selectively under inert atmosphere but is not persistent and probably decomposes as in the case of the POZ counterpart.

In the case of DMAC, the spectroscopic investigations of the reaction mixture revealed the growth of low‐intensity bands at ca. 525 and 575 nm that can be tentatively ascribed to the aminium species (Figure 2E), compatible with the simulated spectra (Figures S12 and S13). The low intensity of the absorption bands ascribed to DMAC^•+^ is probably due to the higher oxidation potential of this substrate, making the hole transfer unfavorable. Moreover, the nitrogen atmosphere assigned for the selective evolution of aminyl radical did not cause any change in the absorption profile of the mixture attributable to this species (Figure 2F), suggesting that the proton transfer from DMAC^•+^ is strongly inhibited by the low concentration of aminium intermediate in comparison with the POZ and PTZ counterpart. In all three cases, it is slightly evident also the formation of the corresponding radical cations (as minor components of the reaction mixture) under anaerobic conditions as starting intermediates for the aminyl evolution.

In the case of CBZ, no evolution of species was observed in accordance with the thermodynamic constraints, in line with the absence of accessible radical intermediates within the optical window (Figure S14).

Hence, the unsymmetrical CDC product (POZ‐dimer, including the C─N bond) would be expected to be forged only if both (aminyl and aminium) intermediates are simultaneously present in the reaction mixture, as also indirectly suggested by Son and coworkers [34], not observing reactivity of selectively obtained aminyl or aminium derivatives of POZ and PTZ, and show the suitable prerequisites to react.

**SCHEME 2:**
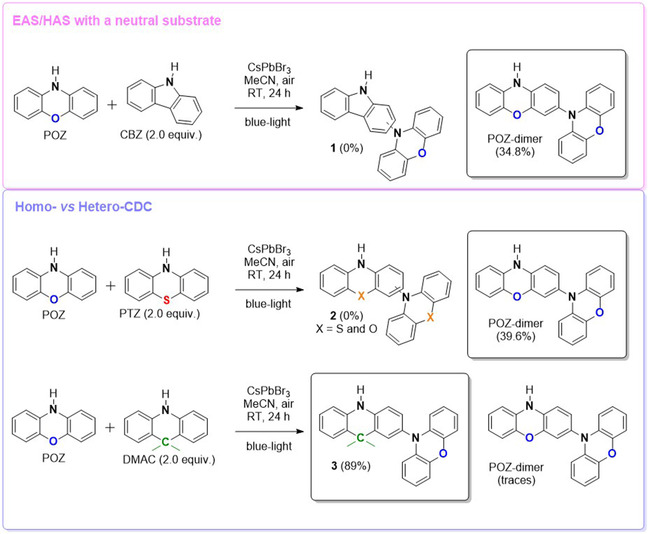
CsPbBr_3_‐photocatalyzed C─N bond formation with different reaction partners. Top: attempted coupling of phenoxazine (POZ) with a neutral substrate (carbazole, CBZ). Bottom: comparison between homo‐ and hetero‐cross‐dehydrogenative coupling (CDC) pathways for POZ with PTZ or DMAC. When the compounds were not isolated, their regiochemistry is not specified.

To further probe the mechanistic scenario, CBZ was introduced as a potential reaction partner for POZ (Scheme 2). CBZ was primarily selected because it exhibits nucleophilic reactivity comparable to that of POZ for structural analogies [18], yet its oxidation potential lies beyond the accessible window of the photocatalyst (Figure 1B), rendering direct photo‐oxidation thermodynamically unfavorable under the reaction conditions. Notably, no hetero cross‐coupling product (**1** in Scheme 2) was detected, while POZ‐dimer was formed in a yield comparable to that reported in entry 7 of Table 1. This outcome effectively excludes a pathway involving electrophilic attack of a POZ‐derived aminium species onto a neutral aromatic partner (CBZ). If such a mechanism were operative, CBZ (despite being redox‐inactive under these conditions) would be expected to intercept the aminium intermediate of POZ. Instead, the exclusive formation of POZ‐dimer therefore supports a mechanism requiring complementary (aminyl and aminium) radical species generated from substrates that both participate in the initial photoinduced charge‐transfer manifold. In fact, the absence of both reactive intermediates in DMAC dimerization de facto precludes the product formation, as experimentally observed, but PTZ was found to be substantially inert in the dimerization process (Scheme 1), notwithstanding the capability of generating both intermediates under aerobic conditions.

To further interrogate the origin of this divergent behavior, competitive experiments were conducted in which PTZ or DMAC was introduced in the presence of a substoichiometric POZ quantity (Scheme 2). This design enabled direct evaluation of whether hetero‐coupling could outcompete the intrinsically favored POZ homodimerization pathway. Under these conditions, PTZ remained entirely unreactive: only POZ‐dimer product was formed and isolated, suggesting that PTZ‐derived intermediates were either not generated in sufficiently high concentrations or were diverted into degradative pathways as non‐reactive species. In striking contrast, DMAC engaged efficiently with POZ, substantially suppressing its homocoupling and delivering the hetero‐CDC product **3** in 89% isolated yield (Scheme 1). DMAC represents a mechanistically informative partner in view of its documented ability to participate in photoinduced charge‐transfer processes exclusively generating the DMAC^•+^ intermediate under aerobic conditions. The selective formation of the cross‐coupling product **3** therefore provided compelling evidence that effective C─N bond formation required a precise electronic complementarity between the interacting radical species, rather than mere accessibility of oxidative activation.

### 2.3 Mechanistic Insights: Theory

To rationalize the divergent reactivity of POZ from PTZ and DMAC, we performed spin‐unrestricted DFT calculations (computational details in the Supporting Information) on the corresponding aminyl and aminium species. Spin density provides a primary descriptor for identifying reactive sites in radical structures; accordingly, Mülliken population analyses were used to compare spin distributions for POZ, PTZ, and DMAC radical intermediates in both forms deduced by spectroscopic evidence.

**FIGURE 3:**
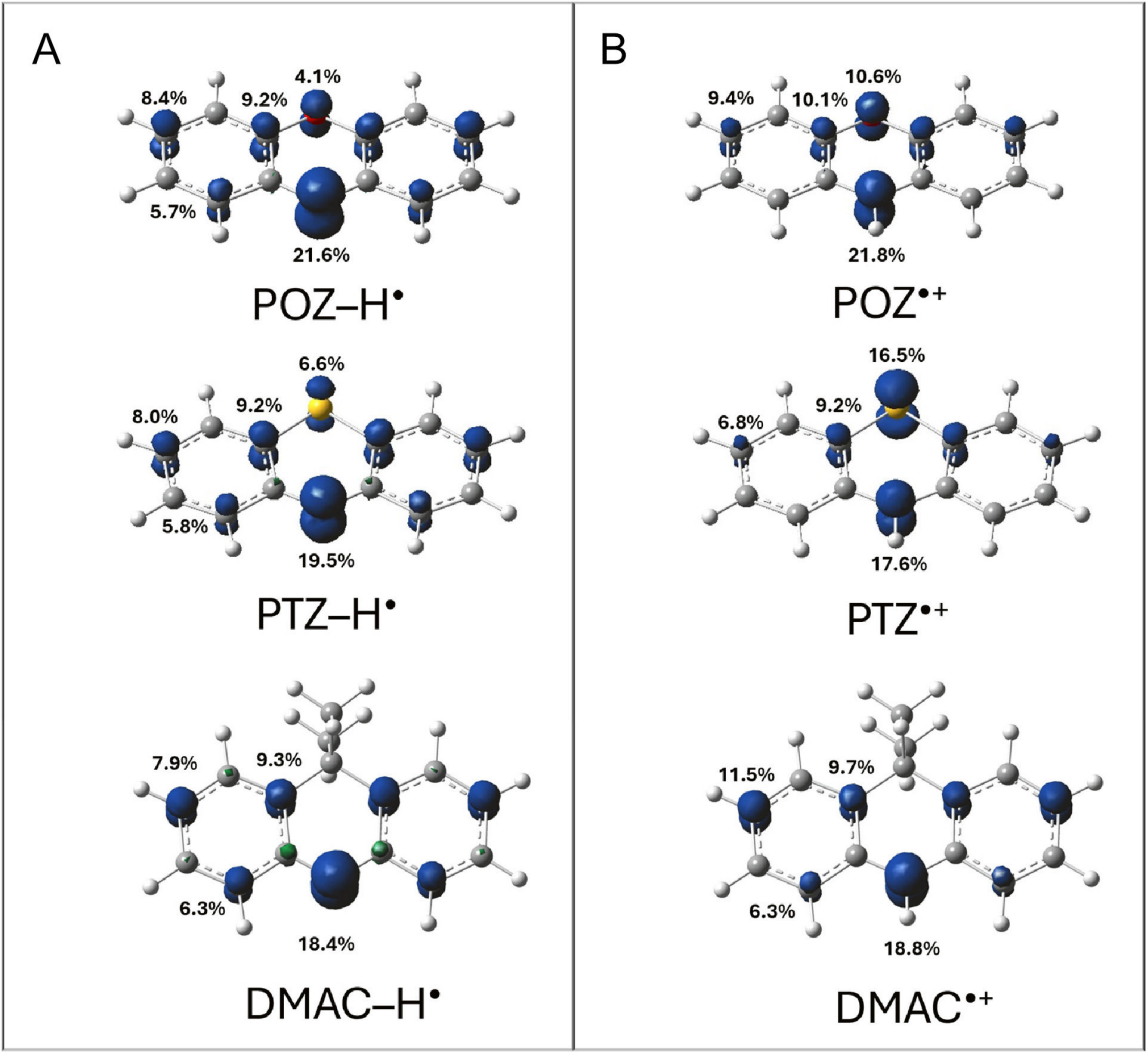
Spin‐density distributions for the aminyl and aminium radicals of POZ and PTZ. Isosurface plots (0.01 e/bohr^3^) for POZ–H^•^, PTZ–H^•^, and DMAC–H^•^ (panel A) and for POZ^•+^, PTZ^•+^, and DMAC^•+^ (panel B) showing the calculated unpaired‐electron density delocalization. Numbers indicate the percentage contribution of the spin population on selected atoms showing highest values.

For the neutral aminyl radicals (POZ–H^•^, PTZ–H^•^, and DMAC–H^•^), the electronic structures are closely related since the unpaired electron density was roughly delocalized over the π framework with a dominant contribution on nitrogen atom (Figure 3A). On the basis of this electronic configuration, N‐centered aminyl radicals (when formed) would be expected to primarily undergo dimerization via N─N bond formation, as the natural reactivity of these species [37]. Nevertheless, aside from the detection of a very low‐intensity GC–MS signal tentatively attributable to the corresponding N─N coupled isomer of POZ, no symmetric dimer could be experimentally obtained in all cases. These experimental/theoretical observations suggest that N─N dimer formation is disfavored under the reaction conditions, likely due to the intrinsic instability of the N─N bond, which readily undergoes homolytic cleavage to regenerate the parent aminyl radicals.

Conversely, clear differences emerge upon comparing the unpaired electron spin density of the radical cations. In POZ^•+^, spin density becomes more compact relative to POZ–H^•^ but remains mainly delocalized over heteroatoms and selected aromatic carbons (Figure 3B). In contrast, PTZ^•+^ features spin density predominantly on heteroatoms (nitrogen and sulfur), with only modest carbon‐centered spin density. In DMAC^•+^, the absence of heteroatom projects spin density mainly on C3 position of the aromatic system, thus explaining its propensity to selectively form product **3** in Scheme 2.

Hence, we considered that the POZ^•+^/POZ–H^•^ (and ideally DMAC^•+^/DMAC–H^•^) complementarity favoring cross‐coupling is due to positive charge delocalization, increasing the electrophilicity of the aminium species in the aromatic carbon containing the highest spin density. Conversely, the electronic mismatch between PTZ^•+^ and PTZ–H^•^ disfavors productive C─N radical–radical coupling, probably promoting deactivation pathway, such as back‐electron transfer or catalyst‐mediated quenching, of the formed radical intermediates.

To examine the fate of POZ^•+^, PTZ^•+^, and DMAC^•+^ generated by photoinduced hole transfer, we evaluated two plausible downstream scenarios for the N─H bond dissociation leading to the corresponding aminyl species: i) interaction of the radical cation with superoxide under aerobic conditions and ii) a surface‐mediated pathway (exemplified with PbBr_6_^4−^ structure). Although the computed free energies should not be interpreted in absolute terms, consistent trends emerge.

**FIGURE 4:**
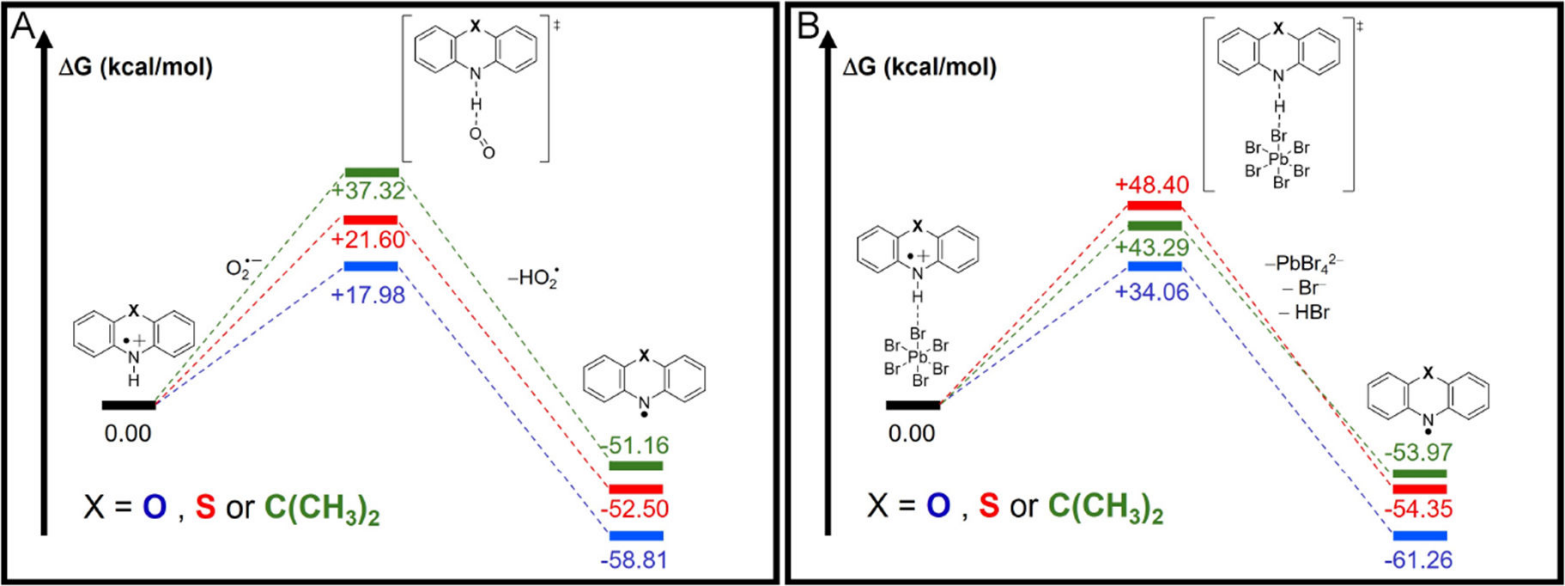
Relative free‐energy profiles highlighting the effect of O, S, and C(CH_3_)_2_ substitution on the substrate scaffold in N─H dissociation. Computed reaction free energies (Δ*G*, kcal/mol) are reported relative to the initial state (set to 0.00) for POZ (blue), PTZ (red), and DMAC (green) analogs along the two pathways promoted by superoxide (A) and PbBr_6_^4−^ (B). Energy diagrams are not on the scale for more evident information deductions.

Along the superoxide‐assisted pathway, POZ^•+^ proceeds through a transition state at 17.98 kcal/mol, whereas PTZ^•+^ requires 21.60 kcal/mol (Figure 4). In the case of DMAC^•+^, the energy barrier was found to be higher (37.32 kcal/mol). The same process simulated for neutral POZ afforded an energy barrier of 63.63 kcal/mol, suggesting that the generation of aminyl species directly from the substrate is an unfavorable process. For the surface‐mediated process, the POZ^•+^ system crosses a transition state at 34.06 kcal/mol, while the PTZ^•+^ analog must overcome a substantially higher barrier (48.40 kcal/mol), slightly lower than that calculated for DMAC^•+^ (43.29 kcal/mol). Thermodynamically, the energies of final states for all species suggest the irreversibility of the process.

An important descriptor to rationalize this behavior may be the acidity of the N─H bond in the photogenerated radical cations: the higher electronegativity of oxygen in POZ can facilitate proton transfer along the reaction coordinate, whereas the PTZ and DMAC analogs are less acidic and therefore less predisposed to evolve along the same pathway.

### 2.4 Mechanistic Insights: Conclusions

Therefore, the calculations support the assumption that the different behavior between POZ and PTZ/DMAC relatives arises from the distinct electronic structures of their radical cations and energy barrier leading to the corresponding neutral radicals (being N‐centered configured in all cases). In the POZ system, the complementary combination of a spin‐delocalized neutral radical and a charge‐delocalized radical cation favors intermolecular coupling between nitrogen‐ and carbon‐centered sites, leading selectively to C─N bond formation at C3 position of POZ scaffold. In contrast, the heteroatom‐centered character of PTZ^•+^ disrupts this balance, providing an electronic rationale for the experimentally observed suppression of PTZ‐dimerization under the proposed photoredox conditions. The high energy barrier for the DMAC–H^•^ evolution along with the thermodynamic limitation in generating DMAC^•+^ species explains the obstacle to the DMAC‐dimer formation. At the same time, theoretical calculations do not preclude the reactivity of these intermediates (PTZ–H^•^ and DMAC^•+^) under the suitable reaction conditions.

**FIGURE 5:**
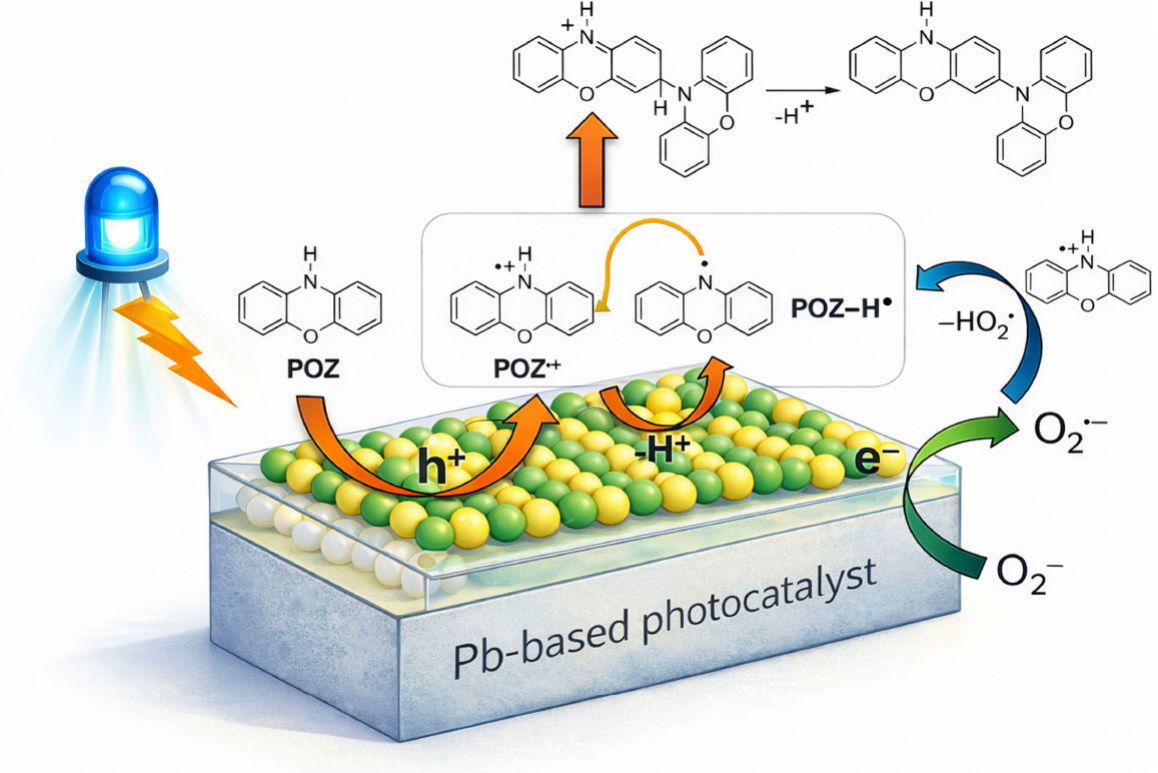
Proposed photocatalytic mechanism for the oxidative dimerization of POZ under blue light irradiation highlighting the role of the experimentally detected intermediates.

On this basis, we can formulate the reaction pathway reported in Figure 5 rationalizing the POZ‐dimer formation. Upon blue LED irradiation, the Pb‐based photocatalyst undergoes photoexcitation, generating electron–hole pairs at the surface. The photogenerated holes are sufficiently oxidizing to activate POZ, leading to the formation of the corresponding radical cation (POZ^•+^). This species can subsequently engage in a proton‐coupled electron transfer process to afford the complementary aminyl radical (POZ–H^•^). The coexistence of these two radical intermediates enables a radical–radical POZ^•+^/POZ–H^•^ coupling event, affording the C─N bonded dimeric intermediate, which undergoes final deprotonation with aromatization to yield the observed biphenoxazine product. In parallel, photogenerated electrons are efficiently quenched by molecular oxygen, resulting in the formation of superoxide (O_2_^•−^) and preventing charge recombination. To rationalize the strict dependence of reactivity on aerobic conditions, it can be also considered the proton abstraction from POZ^•+^ by the superoxide acting as the base to generate the corresponding aminyl radical species.

### 2.5 Intercepting Aminyl Radicals to Forge C─N Bonds

**SCHEME 3:**
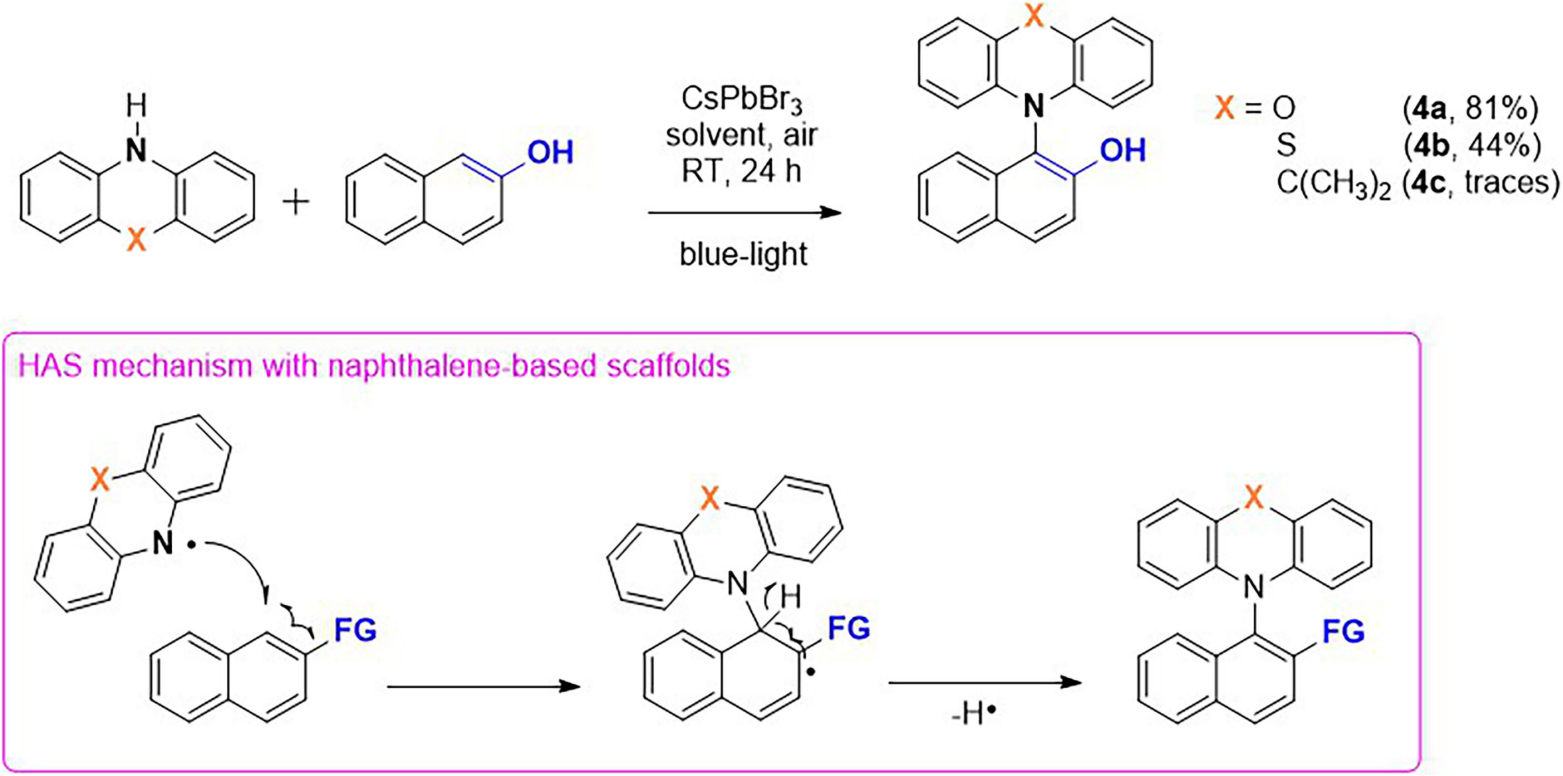
Top: photoinduced cross‐coupling between POZ (PTZ or DMAC) and β‐naphthol. Bottom: general cross‐coupling pathway involving aminyl radicals and naphthalene scaffolds including functional groups capable of stabilizing adjacent radicals.

Having established that photogenerated aminyl species are key intermediates for building C─N bonds, we next investigated whether these species could be intercepted by suitable aromatic partners to enable hetero‐cross‐dehydrogenative coupling. In particular, we sought substrates capable of reacting faster with the aminyl radical than the competing homodimerization of the phenoxazine scaffold. Previous photocatalytic studies have shown that naphthalene‐derived substrates readily participate in radical coupling reactions under photooxidative conditions [38] and β‐naphthol was therefore selected as a model partner of POZ. Under the optimized conditions, irradiation of POZ in the presence of β‐naphthol and CsPbBr_3_ microcrystals afforded the corresponding cross‐coupling product **4a**, while POZ homodimerization was detected only in trace amounts by GC–MS analysis. The product was isolated in 81% yield after chromatographic purification and fully characterized by ^1^H and ^13^C NMR spectroscopy (see Supporting Information), allowing unambiguous assignment of its regiochemistry (Scheme 3).

To further probe the mechanistic scenario, the same transformation was examined using PTZ as the aminyl precursor. Under identical reaction conditions, the corresponding cross‐coupled product **4b** was obtained in 44% yield (Scheme 3). This reduced efficiency is coherent with the lower propensity of PTZ to evolve toward the corresponding aminyl intermediate under the operative photoredox conditions (vide supra). In contrast, DMAC failed to engage in productive coupling with β‐naphthol owing to absence of photogenerated aminyl species. Taken together, these observations further argue against a mechanism involving electrophilic aromatic substitution by an aminium species.

Electrochemical and spectroscopic investigations provide additional support for this interpretation. Cyclic voltammetry and UV–vis monitoring indicated that direct photooxidation of β‐naphthol by CsPbBr_3_ is not thermodynamically accessible under the reaction conditions, and no spectroscopic evidence for β‐naphthol‐derived radical intermediates was therefore observed (Figures S15 and 16). Accordingly, product formation is best rationalized through a homolytic aromatic substitution (HAS) pathway. In this scenario, the aminyl radical (POZ–H^•^) adds to the naphthol ring to generate a resonance‐stabilized cyclohexadienyl radical, which subsequently undergoes hydrogen‐atom removal and rearomatization to afford the observed C─N coupled product (Scheme 3).

Catalyst recyclability was also examined in the model cross‐dehydrogenative coupling between POZ and β‐naphthol. After each run, the CsPbBr_3_ microcrystals were recovered by filtration, washed, and reused in subsequent experiments. The photocatalyst retained its activity for at least five consecutive cycles, affording the cross‐coupled product with only a marginal decrease in isolated yield (81% to 78% after five cycles). Diffuse reflectance spectroscopy (DRS) analysis of the recovered catalyst after the recycling experiments confirmed that its optical features remained essentially unchanged, indicating preservation of the structural integrity of the perovskite material (Figure S17).

**FIGURE 6:**
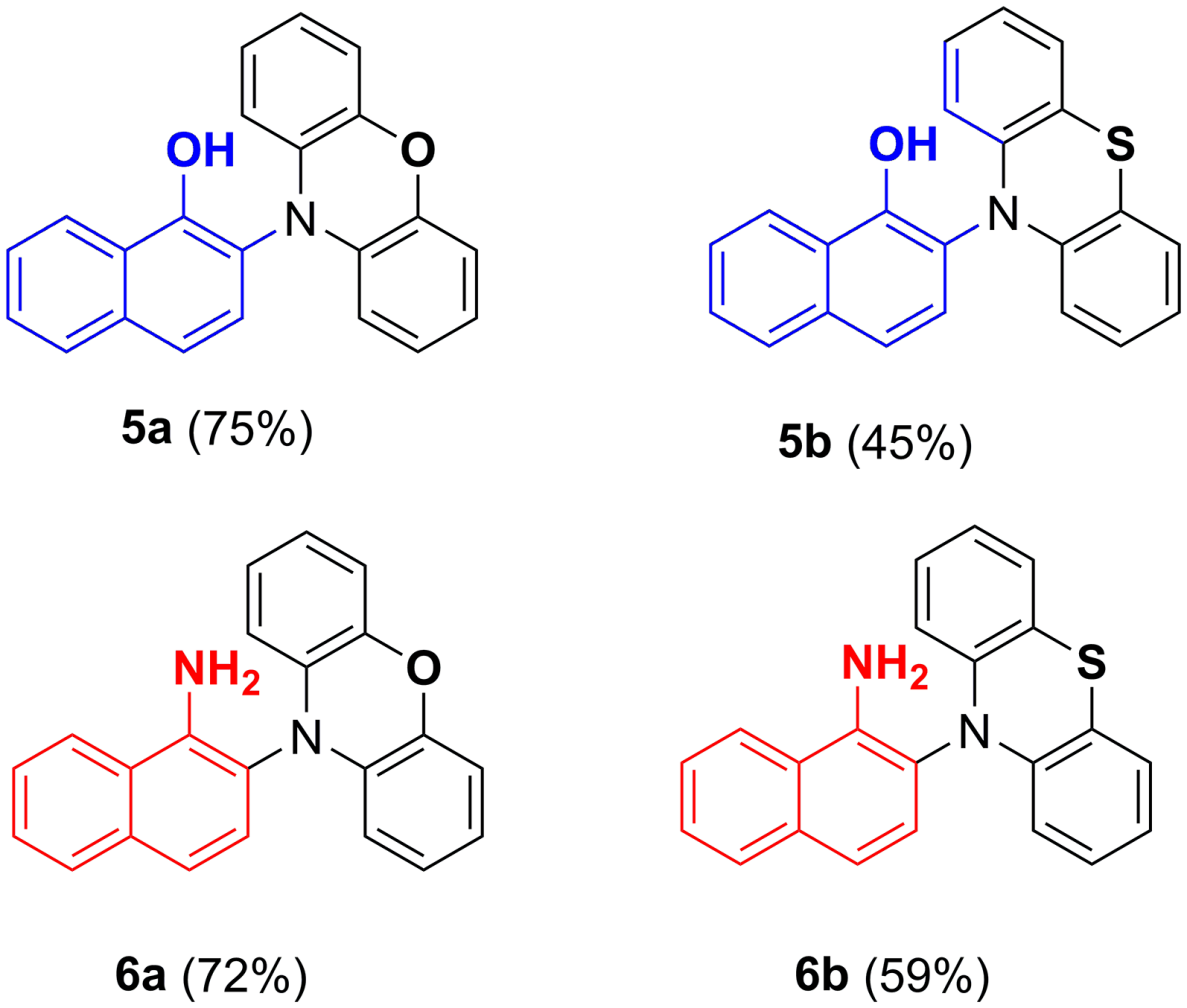
CsPbBr_3_‐photocatalyzed hetero‐cross‐dehydrogenative C─N coupling of POZ and PTZ with naphthalene‐based partners under blue light irradiation. Isolated yields are reported.

Encouraged by these results, we next examined the scope of the reaction by exploring additional aromatic partners capable of intercepting the photogenerated aminyl radicals under the same photocatalytic conditions. 1‐Naphthol and 1‐naphthylamine were selected as representative substrates to demonstrate the feasibility of the photocatalytic hetero‐CDC process with POZ or PTZ, affording the corresponding C**─**N bonded products in good yields (Figure 6). Again POZ‐based products **5a** and **6a** were obtained in higher yields (75% and 72%, respectively) with respect to the PTZ‐based analogs **5b** and **6b** (Figure 6).

By contrast, simpler aromatic substrates lacking an extended conjugated framework were largely ineffective. For example, *p*‐cresol produced only trace amounts of the corresponding cross‐coupled product, while POZ homodimerization remained the dominant reaction pathway. A similar outcome was observed for *p*‐toluidine, where only trace signals attributable to the cross‐coupled product were detected by GC–MS analysis. Control experiments further highlighted the importance of heteroatom substitution in the aromatic partner. When naphthalene itself was subjected to the same reaction conditions in the presence of POZ, no cross‐coupling product was detected. This observation suggests that heteroatoms (such as oxygen or nitrogen) play a crucial role in stabilizing the cyclohexadienyl radical intermediate formed after N‐centered radical addition, thereby lowering the energetic penalty associated with temporary loss of aromaticity (as schematized in Scheme 3). At the same time, efficient interception of photogenerated aminyl radicals requires aromatic partners (naphthalene more effective than benzene) capable of accommodating the transient dearomatization inherent to the homolytic aromatic substitution pathway.

## 3 Conclusions

CsPbBr_3_ microcrystals enabled light‐induced C─N bond formation from aromatic heterocyclic amines under aerobic conditions at room temperature. Among structurally related substrates, phenoxazine uniquely underwent efficient oxidative dimerization, whereas phenothiazine, 9,9‐dimethyl‐9,10‐dihydroacridine, and carbazole displayed suppressed or divergent reactivity. The combined experimental and computational analysis established that productive coupling required the simultaneous presence of complementary radical partners, namely, a charge‐delocalized radical cation and a spin‐delocalized aminyl radical, whose formation and persistence depended sensitively on substrate electronic structure and N─H activation energetics. Spectroscopic interrogation under controlled atmospheres revealed that oxygen was essential to sustain steady‐state radical populations and prevent recombination, while electrochemical measurements defined the accessible redox window of the perovskite photocatalyst. Density functional calculations further demonstrated that subtle differences in spin and charge localization, together with substrate‐dependent barriers for N─H dissociation, governed whether radical intermediates evolved toward coupling or deactivation pathways.

Beyond homodimerization, interception of photogenerated aminyl radicals enabled selective hetero‐cross‐dehydrogenative coupling with electronically competent neutral partners. These results identify electronic complementarity between radical intermediates as a decisive design parameter and establish metal‐halide perovskite microcrystals as versatile semiconductor platforms for controlled N‐centered radical reactivity. Despite the promising reactivity profile, the present system exhibits intrinsic limitations. Productive coupling was observed only for substrates capable of generating complementary radical intermediates within the accessible redox window of CsPbBr_3_ semiconductor. Electronically mismatched heterocycles, although oxidizable, did not evolve toward productive C─N bond formation, highlighting the sensitivity of the process to subtle differences in spin and charge localization. Moreover, neutral aromatic partners required sufficient stabilization of the cyclohexadienyl radical intermediate to outcompete homodimerization of phenoxazine. These observations indicate that both redox compatibility and radical persistence represent critical design parameters for extending the scope of this transformation. While lead‐halide perovskites offer advantageous photophysical and redox characteristics, translating these concepts to environmentally benign semiconductor platforms could be an important objective. More broadly, these findings demonstrate how semiconductor photocatalysts can be used to control complementary radical populations at the catalyst surface, opening new opportunities for designing cross‐dehydrogenative coupling reactions under mild photoredox conditions. Future modulation of perovskite composition and surface chemistry may expand the accessible substrate space and further advance mild oxidative C─N bond construction.

## Supporting Information

Additional supporting information can be found online in the Supporting Information section.

## Funding

Ministero dell’Università e della Ricerca 10.13039/501100021856 (PE0000021, 2022C7Z2RA, P202253ANE).

## Conflicts of Interest

The authors declare no conflicts of interest.

## Supporting information

Supplementary Material

## Data Availability

The data that support the findings of this study are available in the supplementary material of this article.
