# Supplementary material for: Intercepting Photogenerated Aminyl Radicals at Metal‐Halide Perovskite Microcrystals to Forge C─N Bonds With Non‐Preactivated Substrates
Source: Small Sci. 2026 May 18;6(5):e70306. doi: 10.1002/smsc.70306 (PMC13181596; doi:10.1002/smsc.70306)
Supplement: Supplementary file 1 — Supplementary Material [file SMSC-6-e70306-s001.pdf]

Supporting Information

Intercepting Photogenerated Aminyl Radicals at Metal-Halide Perovskite Microcrystals to Forge C–N Bonds with non-Preactivated Substrates

Daniele Conelli[a], Nicola Margiotta[b], Gian Paolo Suranna[a,c] and Roberto Grisorio[a]*

[a]Dipartimento di Ingegneria Civile, Ambientale, del Territorio, Edile e di Chimica (DICATECh), Politecnico di Bari, Via Orabona 4, 70125 Bari, Italy. E-mail: roberto.grisorio@poliba.it

[b]Dipartimento di Chimica, Università degli Studi di Bari “Aldo Moro”, via Orabona 4, 70125 Bari, Italy.

[c]CNR-NANOTEC – Institute of Nanotechnology, c/o Campus Ecoteckne, Via Monteroni, 73100 Lecce, Italy.

Table of Contents

Experimental Section	S3
References	S6
Figures	S7
Computational Data	S24

EXPERIMENTAL SECTION

Materials and methods. All reagents and solvents were purchased from commercial suppliers and used without further purification unless otherwise stated.

UV–Vis diffuse reflectance spectroscopy was conducted using a Jasco V670 spectrophotometer equipped with an integrating sphere.

The substrate conversion and the product evolution were determined by GC–MS (EI, 70 eV) performed on an HP 6890 instrument equipped with an HP-5MS 5% phenyl methyl siloxane (30.0 m × 250 m × 0.25 m) coupled with an HP 5973 mass spectrometer passing from 50 °C to 280 °C with a ramp of 15 °C/min.

1D ^{1}H and ^{13}C NMR and 2D [15N,1H] HSQC spectra were recorded at room temperature on a Bruker Avance III Ultrashield 700 MHz spectrometer equipped with a four-channel CryoProbe (^{1}H, ^{13}C, ^{15}N, ^{31}P). 1H and 13C chemical shifts (δ) are reported in ppm relative to residual solvent signals. 15N spectra were referenced to external standard 1.5 M 15NH4Cl in 1 M HCl set at 23.6 ppm. Multiplicities are reported as s (singlet), d (doublet), t (triplet), dd (doublet of doublets), td (triplet of doublets), and m (multiplet), while coupling constants (J) are reported in Hz

Cyclic voltammetry (CV) measurements were performed in dry acetonitrile containing 0.1 M tetrabutylammonium hexafluorophosphate ($n\text{-}Bu_4NPF_6$) as the supporting electrolyte, using a conventional three-electrode configuration. A glassy carbon disk electrode (3 mm diameter) was employed as the working electrode, a platinum wire served as the counter electrode, and a silver wire was used as a pseudo-reference electrode. All measurements were carried out under a nitrogen atmosphere at room temperature. Ferrocene (Fc, 1.0 mM) was added to each solution as an internal standard for potential calibration, and all potentials are reported versus the Fc/Fc^{+} redox couple unless otherwise stated. Oxidation onset potentials were determined from the intersection of the baseline and the tangent drawn at the rising portion of the anodic wave. The electrochemical behaviour of phenoxazine (POZ), phenothiazine (PTZ), carbazole (CBZ), and β-naphthol was investigated under identical conditions to enable direct comparison of their redox accessibility. The resulting voltammograms were used to evaluate the relative oxidation onsets of the substrates and their compatibility with the oxidative window of the $CsPbBr_3$ photocatalyst. To convert the measured onset potentials to the normal hydrogen electrode (NHE) scale, the accepted value of +0.63 V for the Fc/Fc^{+} couple in acetonitrile was employed. On this basis, the

oxidation onset potentials of the investigated substrates were referenced to the NHE scale and compared with the estimated valence-band position of $CsPbBr_3$.

Theoretical calculations were performed using the Gaussian 09 software package. Geometry optimizations were carried out within the framework of density functional theory (DFT) without symmetry constraints at the 6-311G+(d,p) level of the theory. Open-shell species were treated using the unrestricted formalism. Frequency calculations were performed at the same level of theory to confirm the nature of the stationary points and to obtain zero-point and thermal corrections. For the simulation of the UV–vis absorption spectra, time-dependent density functional theory (TD-DFT) calculations were carried out on the optimized geometries of the selected intermediates. The CAM-B3LYP functional was employed for the calculation of vertical excitation energies to reproduce the main spectral features of the radical cation and aminyl species. The simulated spectra were obtained by convolution of the calculated vertical transitions with Gaussian functions. The calculated transitions were used only for qualitative comparison with the experimental UV–vis spectra and for assignment of the main absorption bands. Thermochemistry was carried out using LANL2DZ/6-311G+(d,p) basis sets and the M06-2X functional.

Synthesis and reactions. $CsPbBr_3$ microcrystals were prepared according to a previously reported antisolvent reprecipitation protocol developed in our group.[1] Briefly, the perovskite precursors were dissolved in dimethyl sulfoxide (DMSO) and rapidly injected into ethyl acetate as antisolvent, affording $CsPbBr_3$ microcrystals. The resulting material has been fully characterized in our previous studies and was employed in the present work without further modification. In a typical experiment, $CsPbBr_3$ microcrystals (10 mg) were dispersed in acetonitrile (3 mL) in a glass vial open to air. POZ or PTZ (0.10 mmol) was then added. For homocoupling reactions, only the amine substrate was employed, whereas for heterocoupling reactions DMAC, naphthols or 1-naphthylamine (0.20 mmol) were added as coupling partner. The reaction mixture was irradiated with blue LED light (4 W) at room temperature under ambient air, without exclusion of oxygen, while being magnetically stirred. After the indicated reaction time, the mixture was filtered to remove the solid photocatalyst, and the resulting filtrate was subjected to further analysis or purification. For homocoupling reactions, conversion, yield, and selectivity were determined by gas chromatography–mass spectrometry (GC–MS) using biphenyl as an internal standard. Calibration curves were established for quantitative

determination of product yields. For heterocoupling reactions, the crude reaction mixture was purified by column chromatography on silica gel using a hexane/ethyl acetate (9:1, v/v) eluent to afford the corresponding C–N coupled products. Radical scavenger experiments were performed according to the general photocatalytic procedure described above. When required, 1,4-benzoquinone (BQ, 2.0 equivalents relative to the amine substrate) or 2,2,6,6-tetramethylpiperidine-1-oxyl (TEMPO, 10.0 equivalents) was added prior to light irradiation. After completion of the reaction, the photocatalyst was removed by filtration, and the resulting filtrates were analysed by GC–MS using biphenyl as an internal standard.

10H-3,10'-biphenoxazine (POZ-dimer): off-white solid, 35.1% yield. ^{1}H NMR (700 MHz, DMSO-d_6) δ 8.52 (s, 1H), 6.76 (td, *J* = 7.8, 1.9 Hz, 1H), 6.72 (dd, *J* = 8.2, 2.2 Hz, 1H), 6.71 – 6.56 (m, 10H), 6.50 (d, *J* = 7.5 Hz, 1H), 6.02 (dd, *J* = 7.7, 1.2 Hz, 2H) ppm. ^{13}C NMR (176 MHz, DMSO-d_6) δ 144.88, 143.56, 142.77, 134.49, 133.37, 132.29, 130.47, 126.23, 124.76, 124.21, 121.73, 121.29, 117.19, 115.61, 115.27, 113.95, 113.64 ppm. 15N (70.95 MHz; DMSO-d6): 58.32 ppm.

10-(9,9-dimethylacridin-10-yl)phenoxazine (3): deep orange solid, 89% yield, ^{1}H NMR (700 MHz, acetone-d_6) δ 8.47 (s, 1H), 7.43 (d, *J* = 6.9 Hz, 1H), 7.37 (d, *J* = 2.2 Hz, 1H), 7.18 – 7.07 (m, 2H), 7.05 (dd, *J* = 8.3, 2.2 Hz, 1H), 6.99 – 6.80 (m, 2H), 6.73 – 6.65 (m, 3H), 6.64 – 6.62 (m, 3H), 6.00 – 5.94 (m, 2H), 1.59 (s, 6H) ppm. ^{13}C NMR (176 MHz, acetone-d_6) δ 143.85, 139.29, 138.68, 135.03, 131.39, 130.36, 128.54, 128.16, 127.60, 126.85, 125.42, 123.50, 120.99, 120.55, 115.71, 115.05, 113.70, 113.31, 36.23, 30.31 ppm.

1-(phenoxazin-10-yl)naphthalen-2-ol (4a): off-white solid, 81% yield, ^{1}H NMR (700 MHz, DMSO-d_6) δ 10.49 (s, 1H), 7.97 (d, *J* = 8.9 Hz, 1H), 7.94 (d, *J* = 8.1 Hz, 1H), 7.67 (d, *J* = 8.4 Hz, 1H), 7.43 (t, *J* = 7.6 Hz, 1H), 7.36 (m, 2H), 6.72 (d, *J* = 7.9 Hz, 2H), 6.61 (t, *J* = 7.6 Hz, 2H), 6.52 (t, *J* = 7.6 Hz, 2H), 5.58 (d, *J* = 7.9 Hz, 2H) ppm. ^{13}C NMR (176 MHz, DMSO-d_6) δ 153.83, 143.89, 133.28, 132.21, 131.00, 129.58, 129.27, 128.10, 124.20, 124.05, 121.72, 121.31, 119.74, 115.66, 115.00, 112.91 ppm.

1-(phenothiazin-10-yl)naphthalen-2-ol (4b): pale yellow solid, 44% yield: ^{1}H NMR (700 MHz, DMSO-d_6) δ 10.59 (s, 1H), 7.97 (d, *J* = 9.0 Hz, 1H), 7.90 (dd, *J* = 14.3, 8.3 Hz, 2H), 7.53 – 7.37 (m, 2H), 7.37 – 7.22 (m, 1H), 7.11 – 6.91 (m, 2H), 6.89 – 6.63 (m, 4H), 6.18 – 5.76 (m, 2H)

ppm. ^{13}C NMR (176 MHz, DMSO-d_6) δ 154.16, 142.55, 132.05, 130.88, 129.57, 129.02, 128.15, 127.84, 126.84, 124.15, 123.02, 121.24, 119.29, 118.32, 115.65 ppm.

2-(10H-phenoxazin-10-yl)naphthalen-1-ol (5a): off-white solid, 75% yield. ^{1}H NMR (700 MHz, DMSO-d_6) δ 10.70 (s, 1H), 8.26 (d, J = 7.5 Hz, 1H), 7.76 (d, *J* = 7.7 Hz, 1H), 7.58 – 7.46 (m, 2H), 7.42 (d, *J* = 7.9 Hz, 1H), 7.04 (d, *J* = 7.9 Hz, 1H), 6.74 (d, *J* = 9.0 Hz, 2H), 6.61 (t, *J* = 8.2 Hz, 2H), 6.53 (t, *J* = 7.7 Hz, 2H), 5.64 (d, *J* = 9.1 Hz, 2H) ppm. ^{13}C NMR (176 MHz, DMSO-d_6) δ 154.49, 143.59, 134.62, 131.60, 130.15, 128.05, 126.83, 125.89, 124.78, 124.23, 123.80, 122.74, 121.67, 115.66, 113.49, 109.24 ppm.

2-(10H-phenothiazin-10-yl)naphthalen-1-ol (5b): pale yellow solid, 45% yield. ^{1}H NMR (700 MHz, DMSO-d_6) δ 10.72 (s, 1H), 8.26 (d, *J* = 7.7 Hz, 1H), 7.79 (d, *J* = 7.8 Hz, 1H), 7.58 – 7.37 (m, 4H), 7.07 (d, *J* = 7.9 Hz, 1H), 7.06 – 7.01 (m, 2H), 6.82 – 6.73 (m, 4H), 6.05 – 5.95 (m, 2H) ppm. ^{13}C NMR (176 MHz, DMSO-d_6) δ 154.29, 144.01, 131.81, 130.66, 128.05, 127.76, 127.16, 126.91, 126.63, 125.90, 125.01, 123.69, 122.95, 122.41, 119.03, 115.97 ppm.

2-(10H-phenoxazin-10-yl)naphthalen-1-amine (6a): pale yellow solid, **72%** yield. ^{1}H NMR (700 MHz, DMSO-d_6) δ 8.15 (d, *J* = 9.0 Hz, 1H), 7.85 – 7.58 (m, 1H), 7.41 (m, 2H), 7.23 (d, *J* = 7.9 Hz, 1H), 6.82 (d, *J* = 7.9 Hz, 1H), 6.69 (d, *J* = 7.9 Hz, 2H), 6.58 (td, *J* = 7.8, 1.2 Hz, 2H), 6.53 – 6.46 (m, 2H), 6.05 (s, 2H), 5.63 (dd, *J* = 8.0, 1.1 Hz, 2H) ppm. ^{13}C NMR (176 MHz, DMSO-d_6) δ 146.11, 143.56, 134.79, 131.21, 130.16, 127.49, 125.07, 124.55, 124.20, 123.96, 122.74, 121.63, 121.56, 115.63, 113.41, 108.32 ppm.

2-(10H-phenothiazin-10-yl)naphthalen-1-amine (6b): purple solid, 59% yield. ^{1}H NMR (700 MHz, DMSO-d_6) δ 8.21 (dd, *J* = 5.6, 3.9 Hz, 1H), 7.73 (dd, *J* = 5.6, 4.0 Hz, 1H), 7.47 – 7.38 (m, 2H), 7.34 (d, *J* = 7.9 Hz, 1H), 7.01 (d, *J* = 9.1 Hz, 2H), 6.83 (d, *J* = 7.9 Hz, 1H), 6.79 – 6.71 (m, 4H), 6.17 (s, 2H), 6.07 – 5.94 (m, 2H) ppm. ^{13}C NMR (176 MHz, DMSO-d_6) δ 146.22, 144.28, 131.50, 130.73, 127.70, 127.43, 126.81, 124.81, 124.30, 124.01, 123.79, 123.00, 122.77, 118.90, 115.97, 107.70 ppm.

REFERENCES

[1] D. Conelli, G. K. Grandhi, A. Tewari, et al., “Synergistic Dye/Photocatalyst Interconnections for Activating Efficient Light-Induced Degradation Pathways,” *Journal of Materials Chemistry C* 13, no. 4 (2025): 1769–1783.

Figures

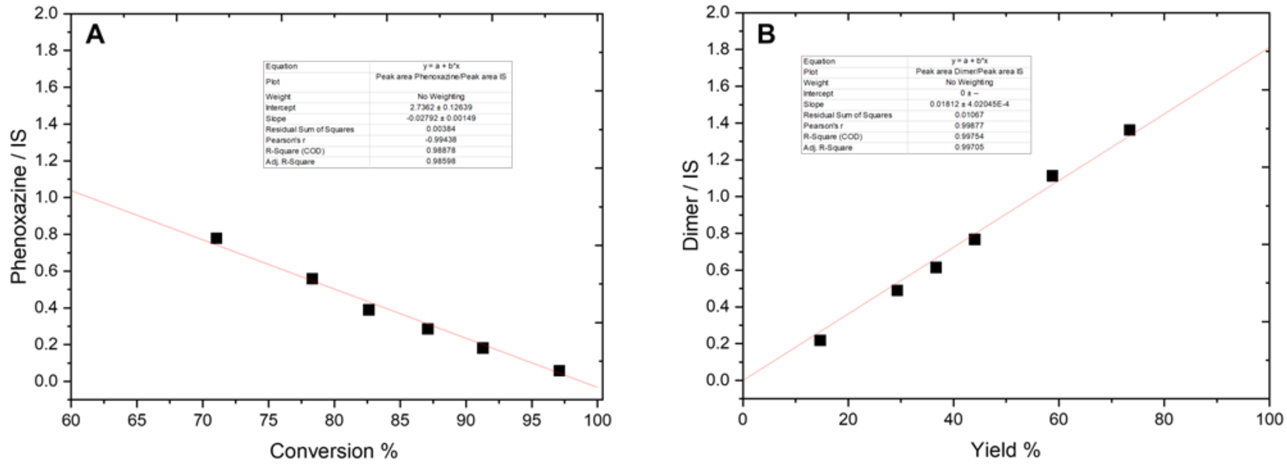

Figure S1. Calibration curves for the GC–MS analyses reported in Table 1 of the manuscript using biphenyl as an internal standard: (A) phenoxazine and (B) phenoxazine dimer, reported as peak area ratios versus conversion and yield, respectively.

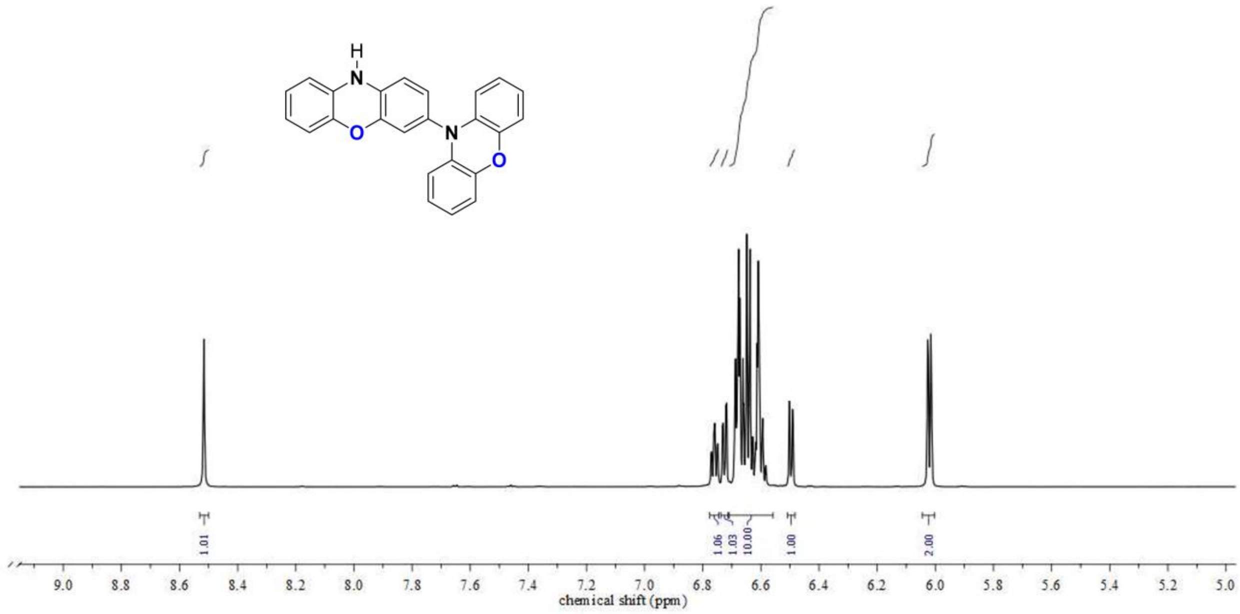

Figure S2. ^{1}H NMR (700 MHz, DMSO-d_6) of 10H-3,10-biphenoxazine (POZ-dimer).

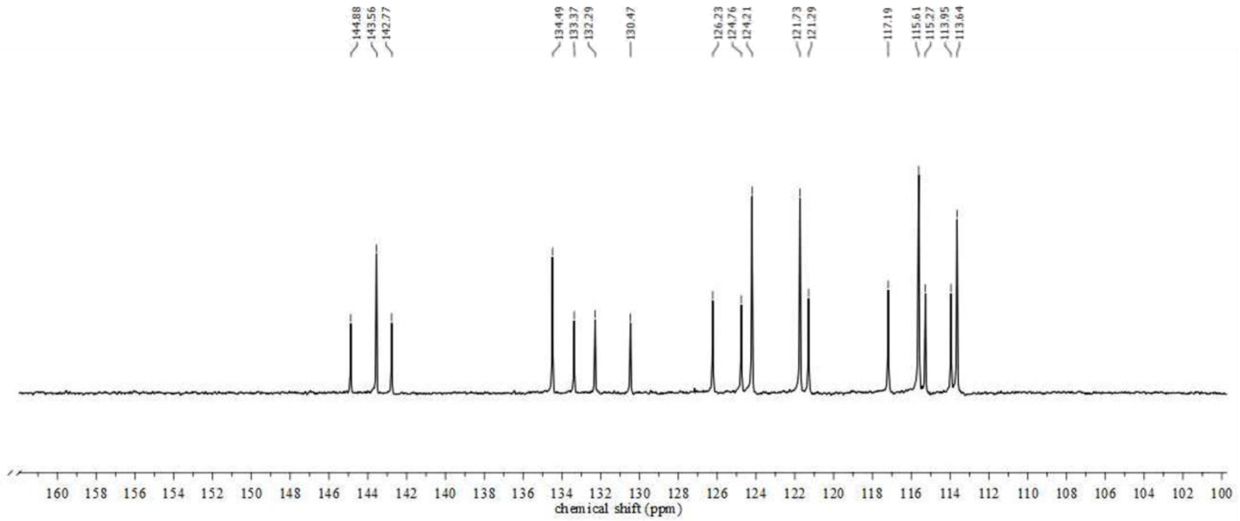

Figure S3. ^{13}C NMR (176 MHz, DMSO-d_6) of 10H-3,10-biphenoxazine (POZ-dimer).

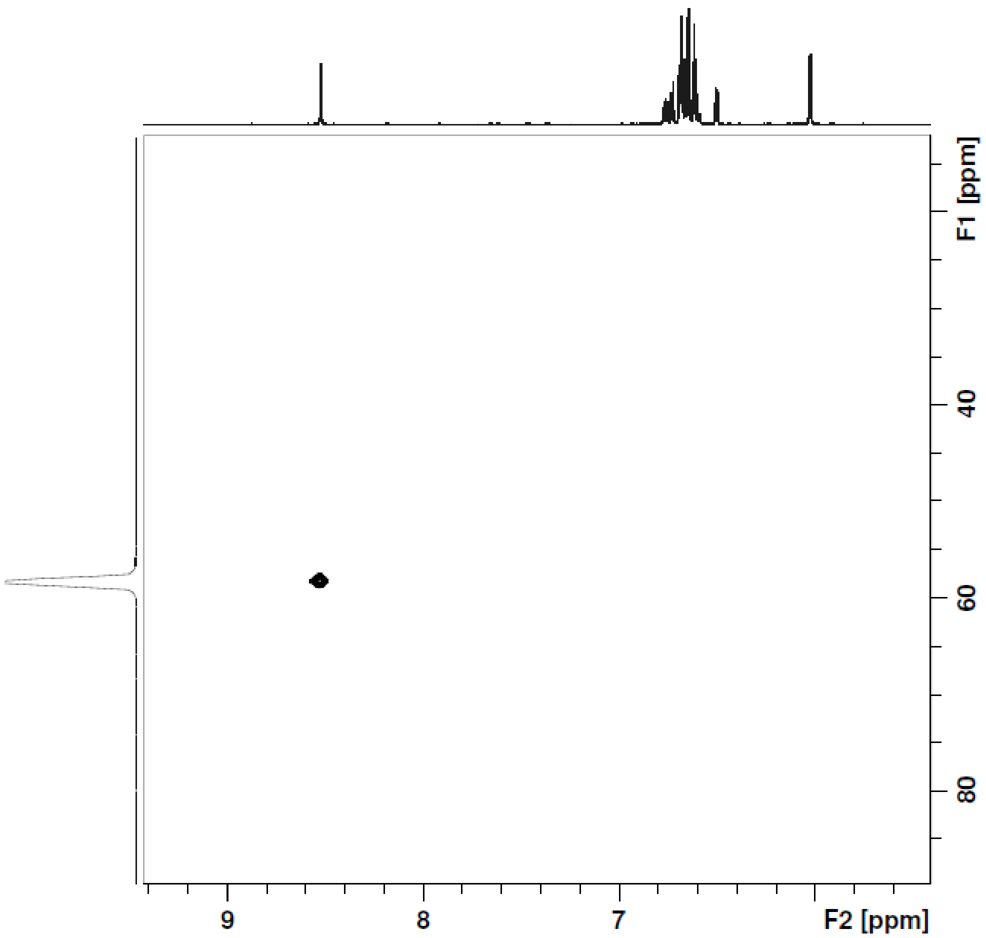

Figure S4. 2D [^{15}N,^{1}H] HSQC NMR (70.95 MHz, ^{15}N; DMSO-d_6) of 10H-3,10′-biphenoxazine (POZ-dimer).

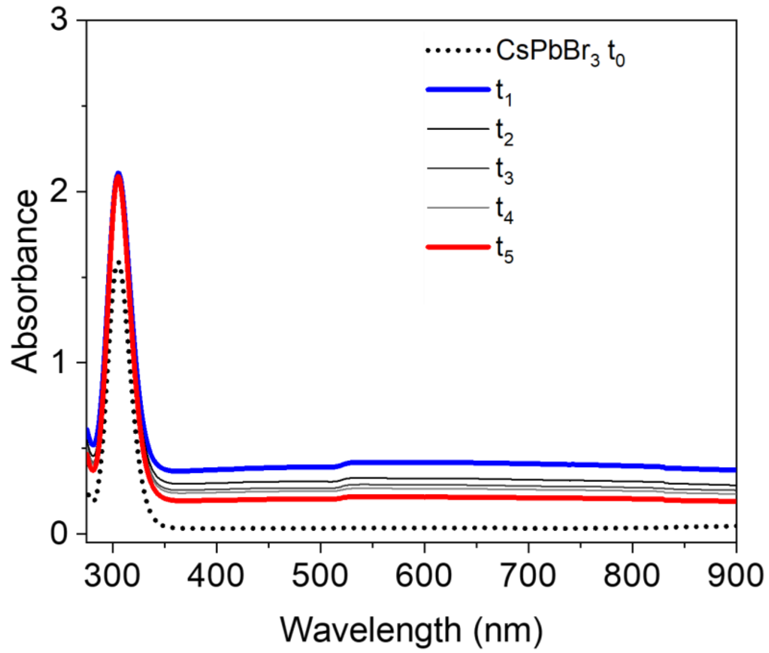

Figure S5. UV–vis absorption spectrum of $CsPbBr_3$ dispersed in MeCN under stirring and monitored at 15 minutes intervals.

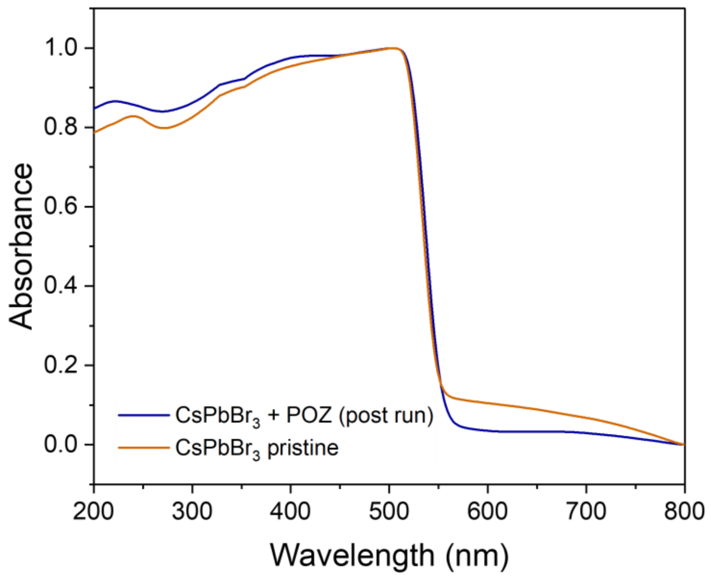

Figure S6. Diffuse reflectance spectra (DRS) of pristine $CsPbBr_3$ and $CsPbBr_3$ recovered after reaction with POZ in the dark.

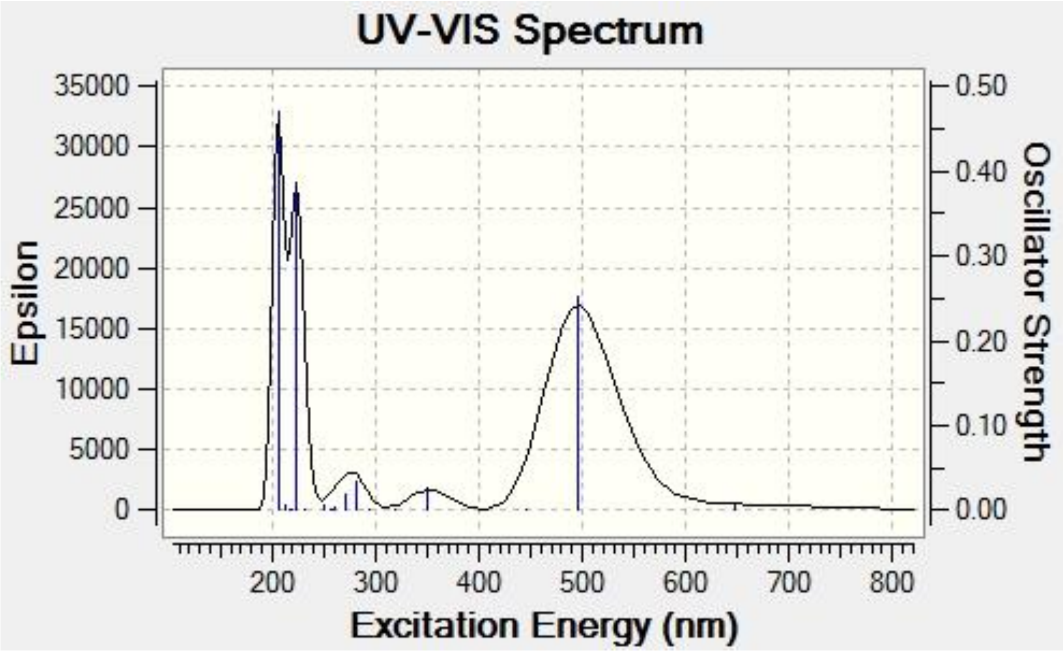

Figure S7. Simulated UV–vis spectrum of $POZ^{\bullet+}$ generated by the corresponding vertical electronic transitions.

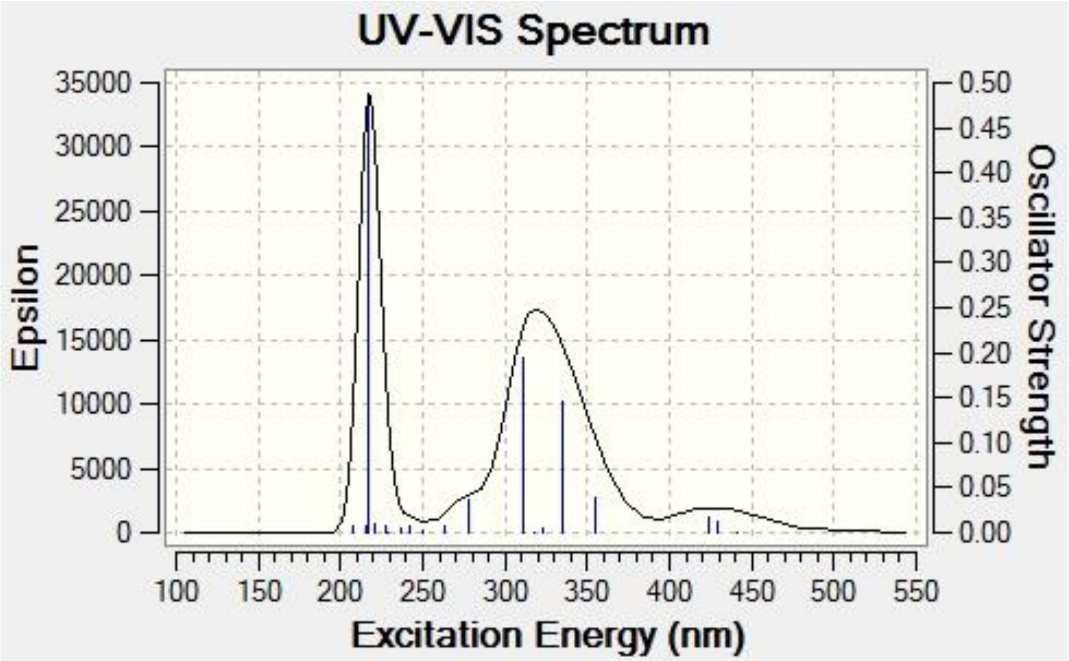

Figure S8. Simulated UV–vis spectrum of the $POZ–H^{\bullet}$ generated by the corresponding vertical electronic transitions.

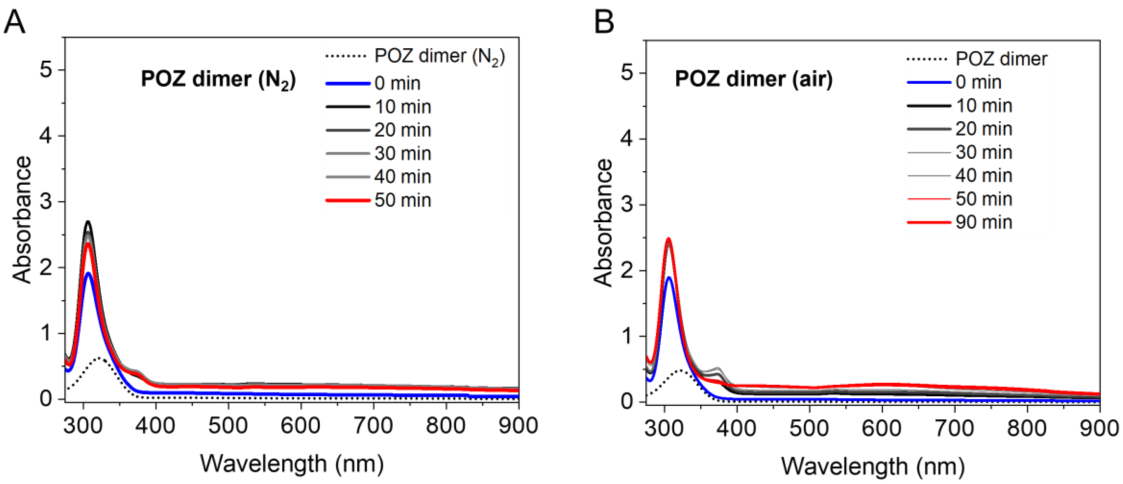

Figure S9. UV–vis absorption spectra of POZ-dimer under the photocatalytic conditions: (A) N_2 atmosphere; (B) air.

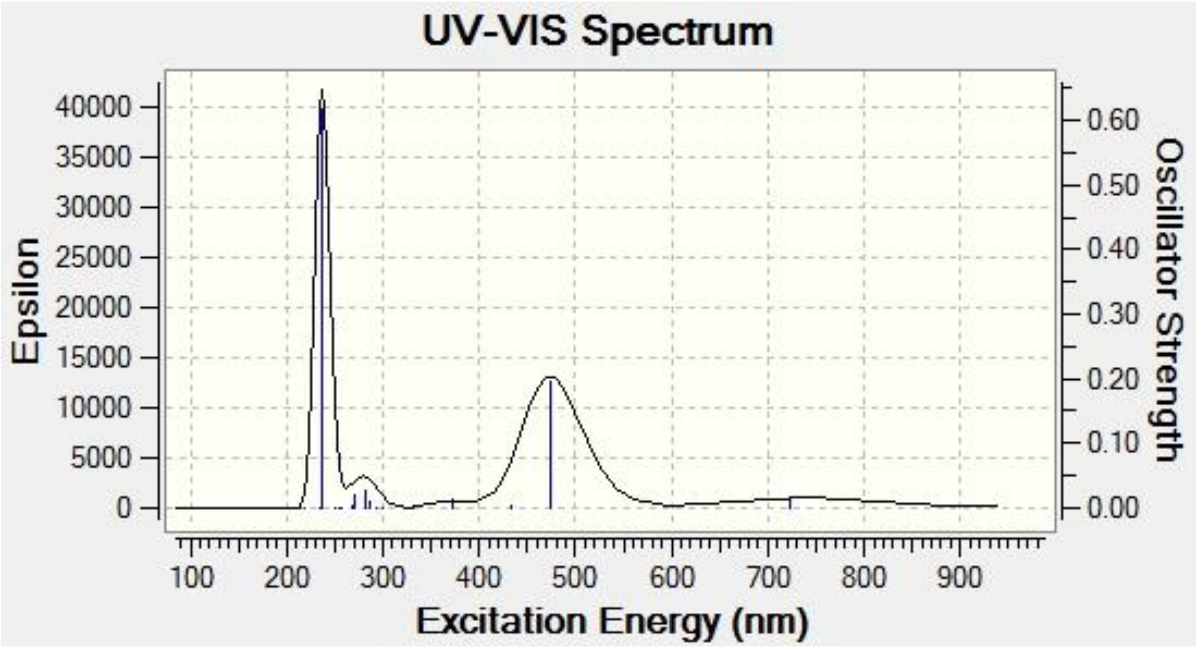

Figure S10. Simulated UV–vis spectrum of $PTZ^{\bullet+}$ generated by the corresponding vertical electronic transitions.

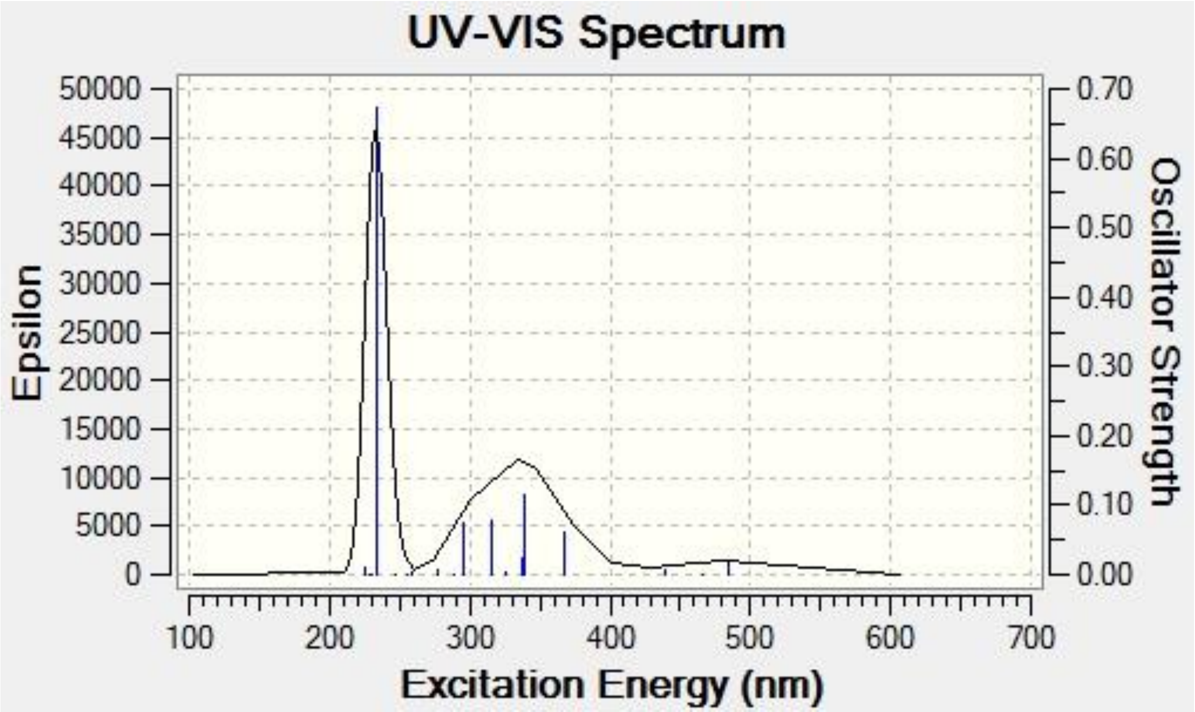

Figure S11. Simulated UV–vis spectrum of the PTZ–H$^{\bullet}$ generated by the corresponding vertical electronic transitions.

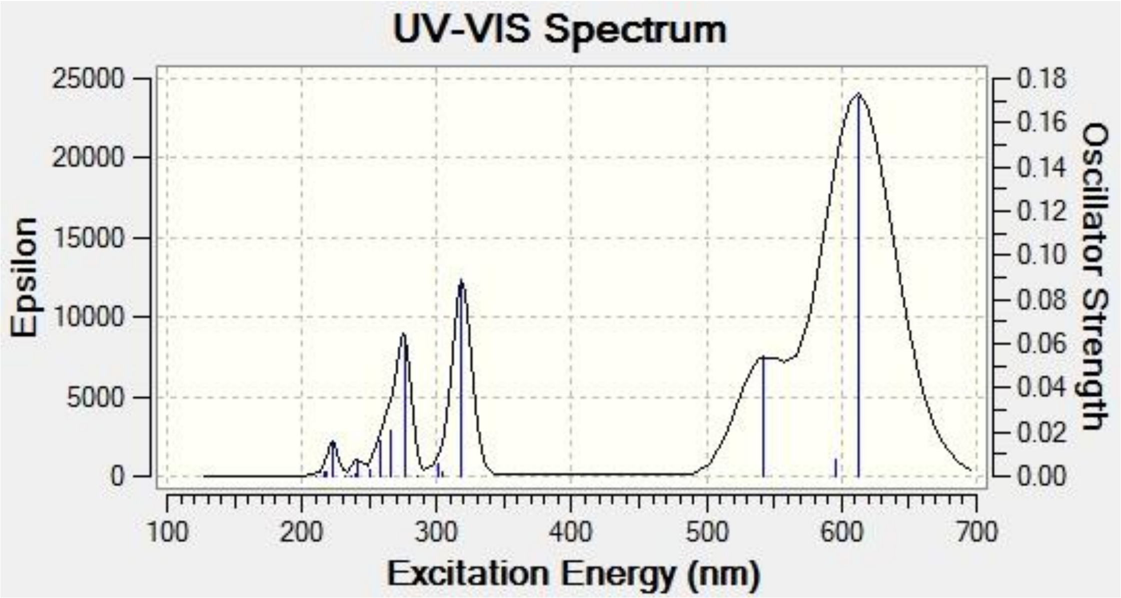

Figure S12. Simulated UV–vis spectrum of DMAC$^{\bullet+}$ generated by the corresponding vertical electronic transitions.

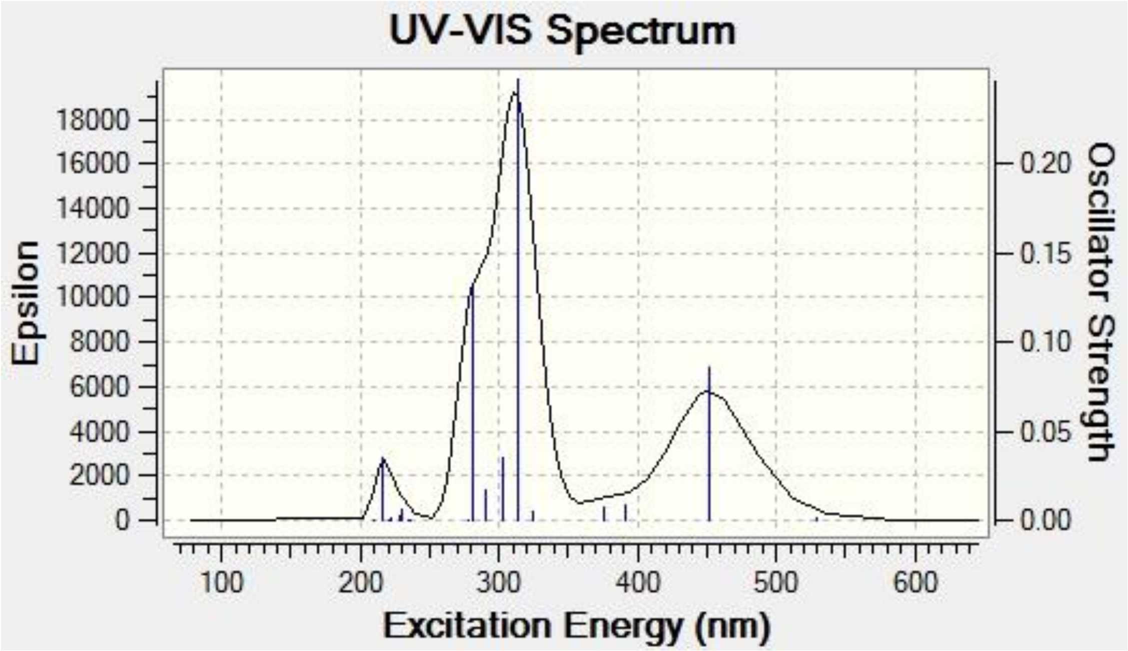

Figure S13. Simulated UV–vis spectrum of the DMAC–H$^{\bullet}$ generated by the corresponding vertical electronic transitions.

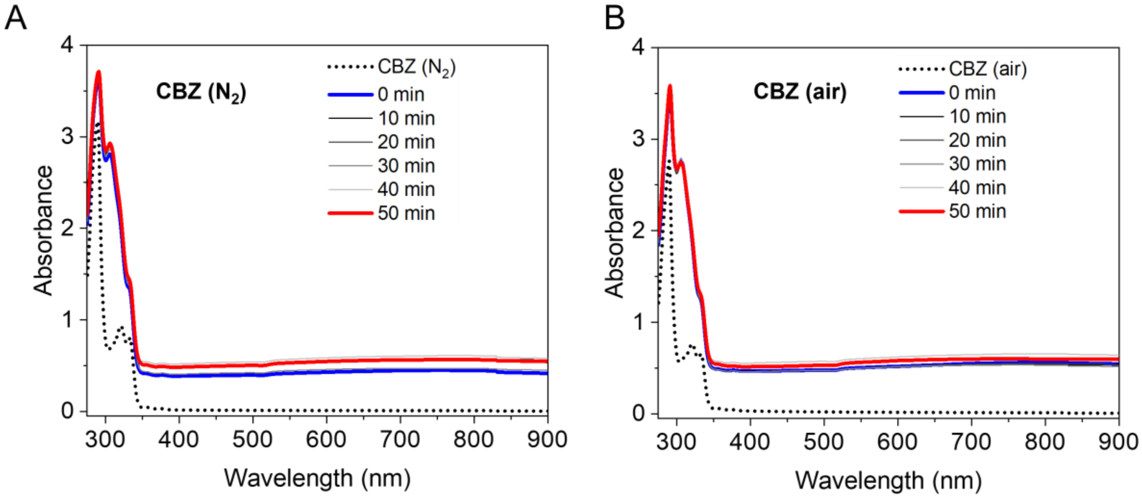

Figure S14. UV–vis absorption spectra of CBZ under the photocatalytic conditions: (A) N_2 atmosphere; (B) air.

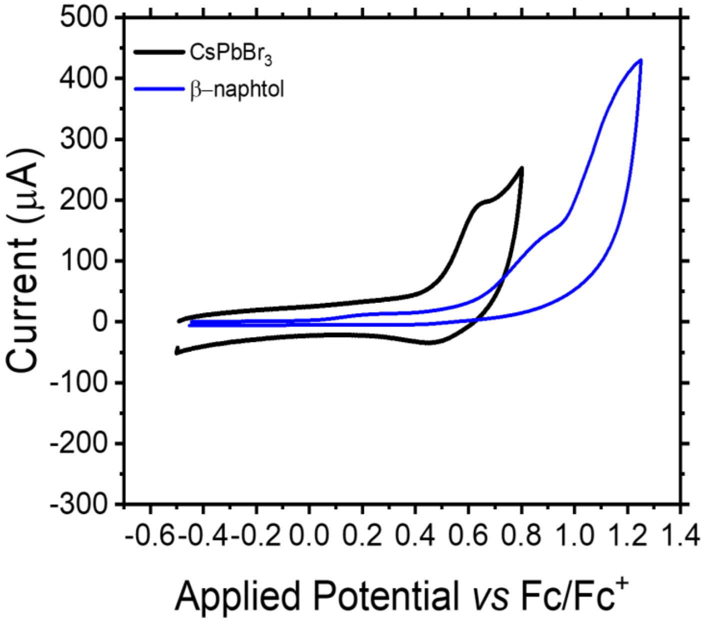

Figure S15. Cyclic voltammetry traces of $CsPbBr_3$ microcrystals and β-naphthol recorded vs Fc/Fc^+.

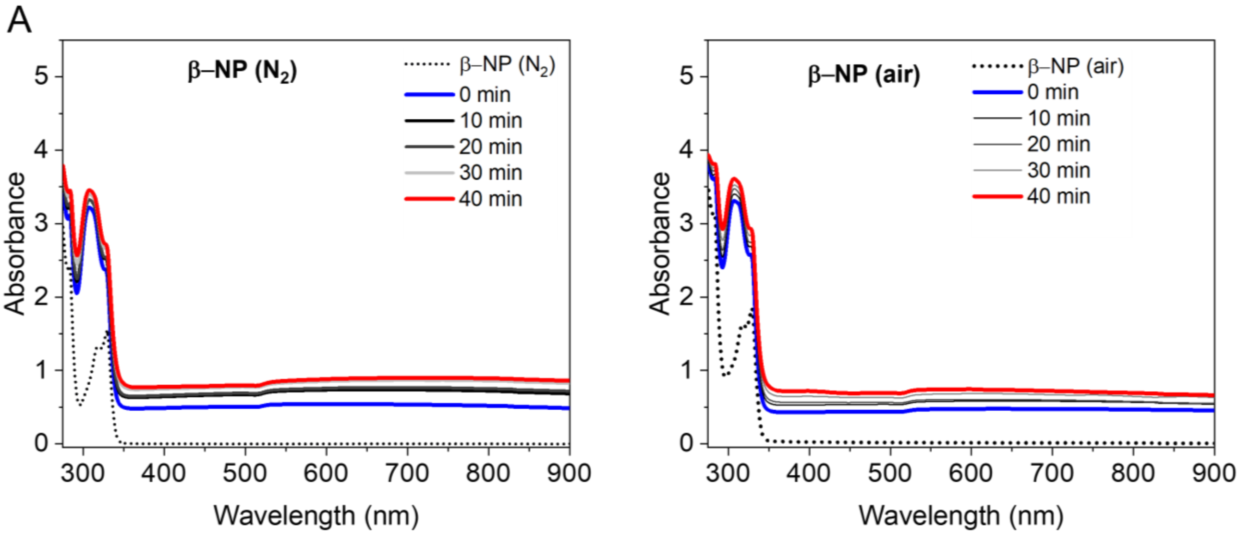

Figure S16. UV–vis absorption spectra of β-naphthol under photocatalytic conditions: (A) N_2 atmosphere, (B) air.

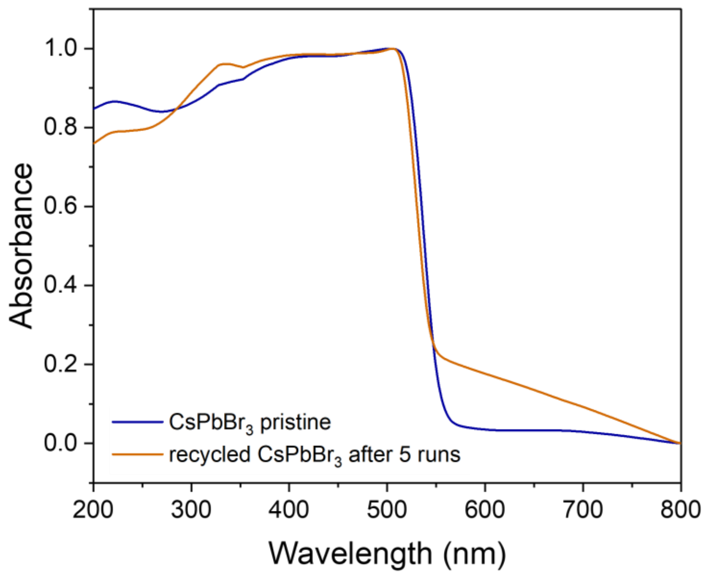

Figure S17. Diffuse reflectance spectra (DRS) of pristine $CsPbBr_3$ and $CsPbBr_3$ recovered after five photocatalytic cycles.

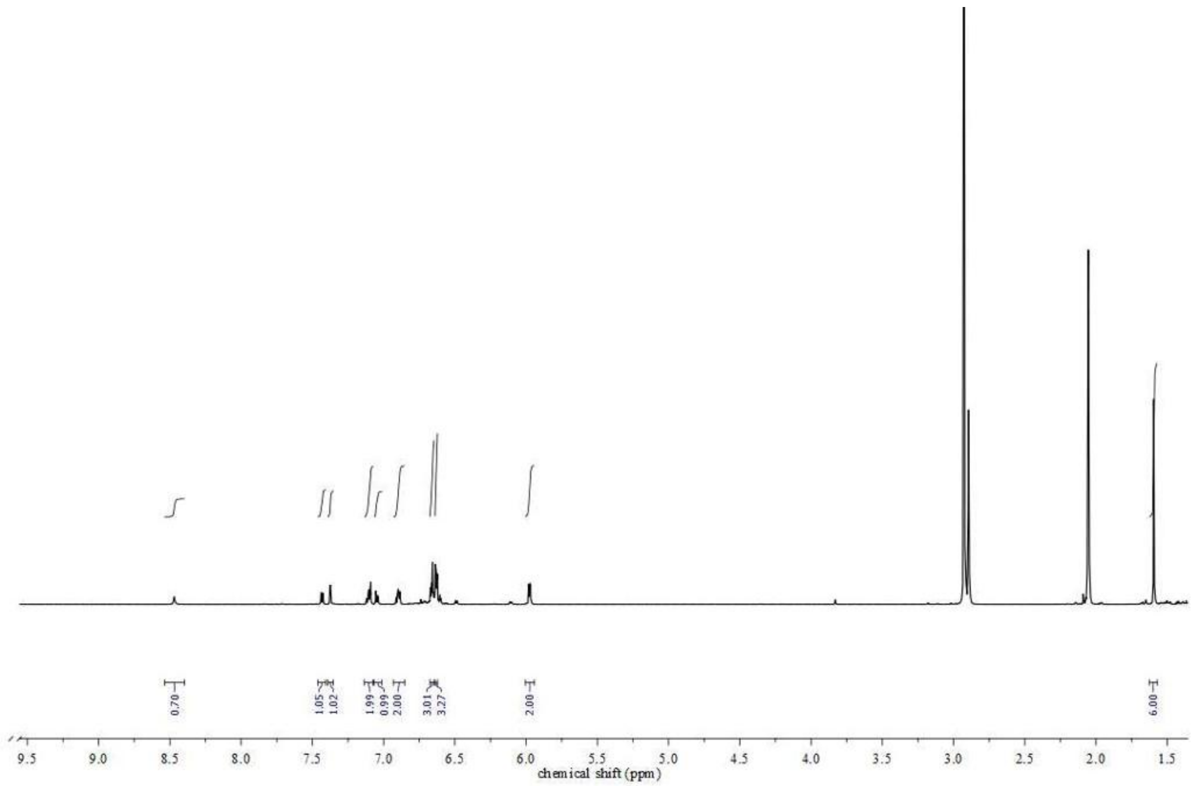

Figure S18. ^{1}H NMR (700 MHz, acetone-d_6) of **3**.

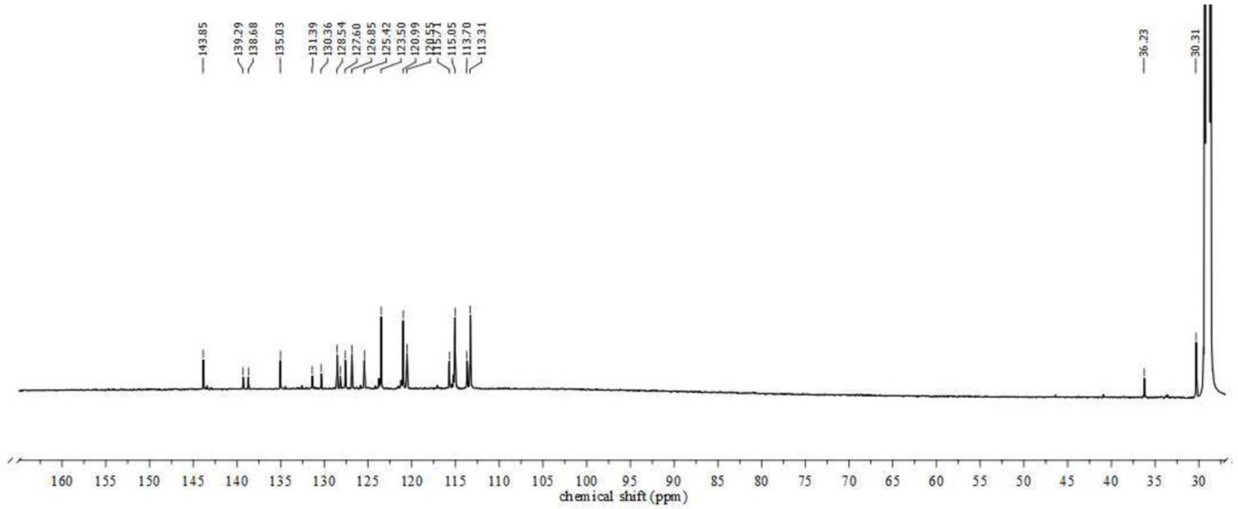

Figure S19. ^{13}C NMR (176 MHz, acetone-d_6) of **3**.

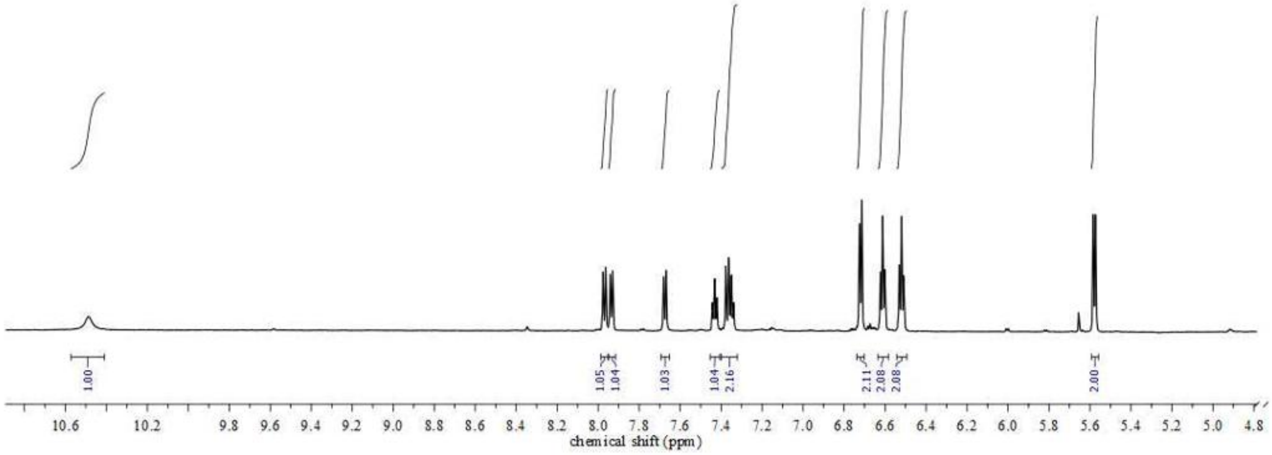

Figure S20. ^{1}H NMR (700 MHz, DMSO-d_6) of **4a**.

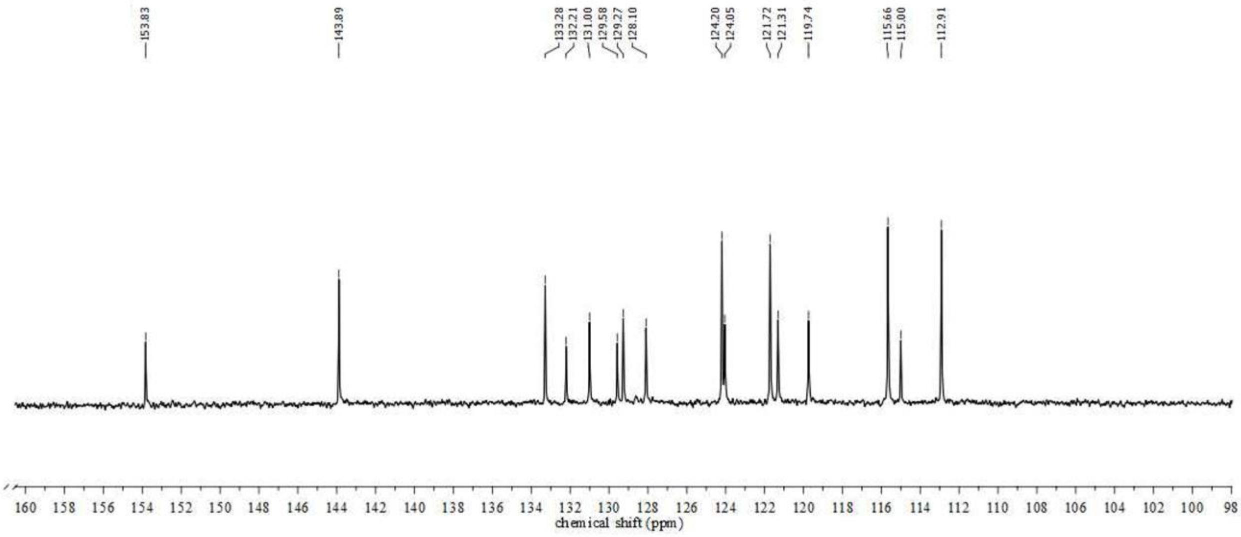

Figure S21. ^{13}C NMR (176 MHz, DMSO-d_6) of **4a**.

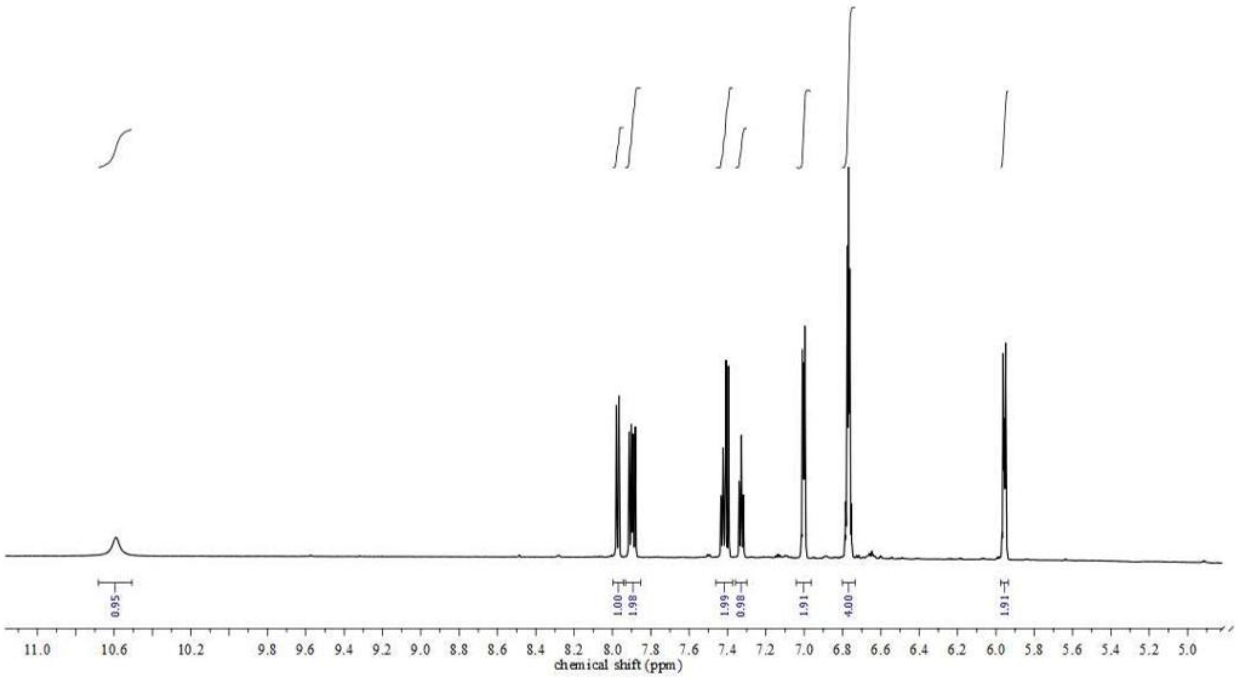

Figure S22. ^{1}H NMR (700 MHz, DMSO-d_6) of **4b**.

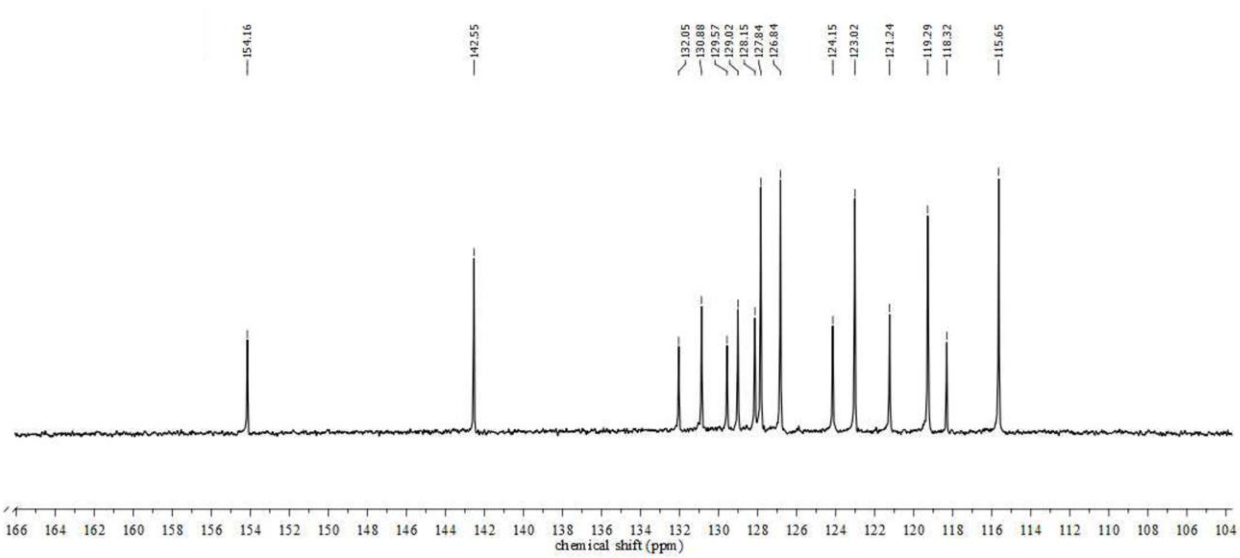

Figure S23. ^{13}C NMR (176 MHz, DMSO-d_6) of **4b**.

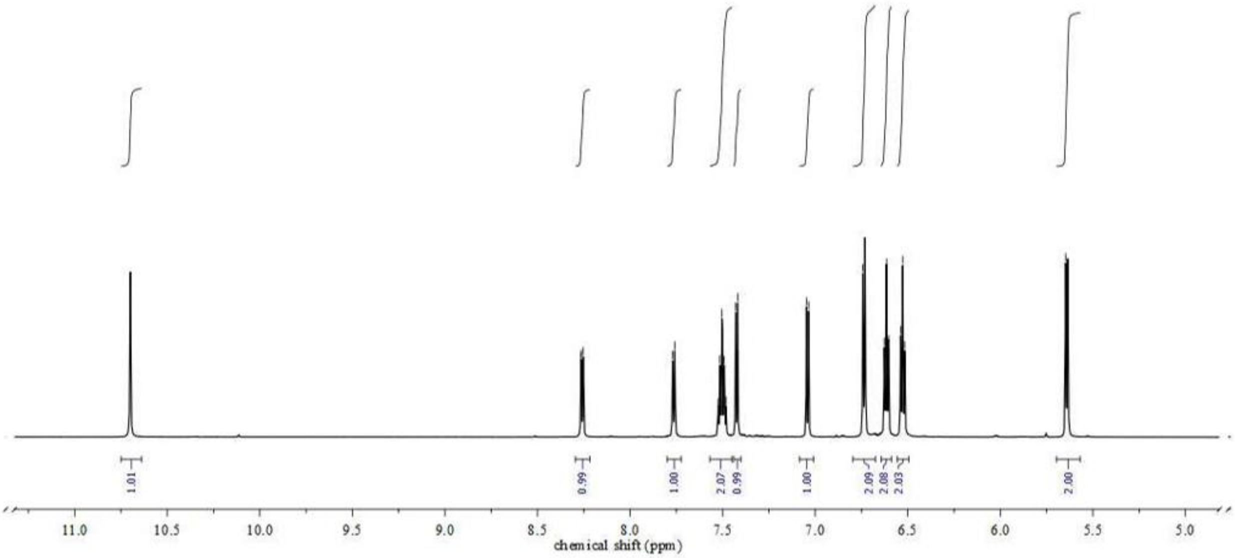

Figure S24. ^{1}H NMR (700 MHz, DMSO-d$_6$) of **5a**.

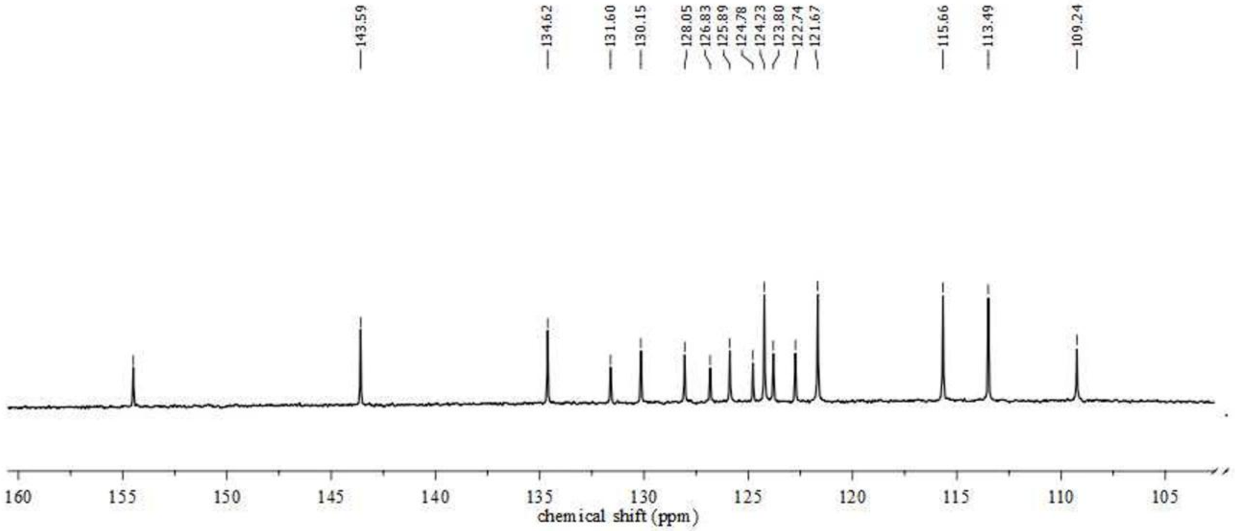

Figure S25. ^{13}C NMR (176 MHz, DMSO-d$_6$) of **5a**.

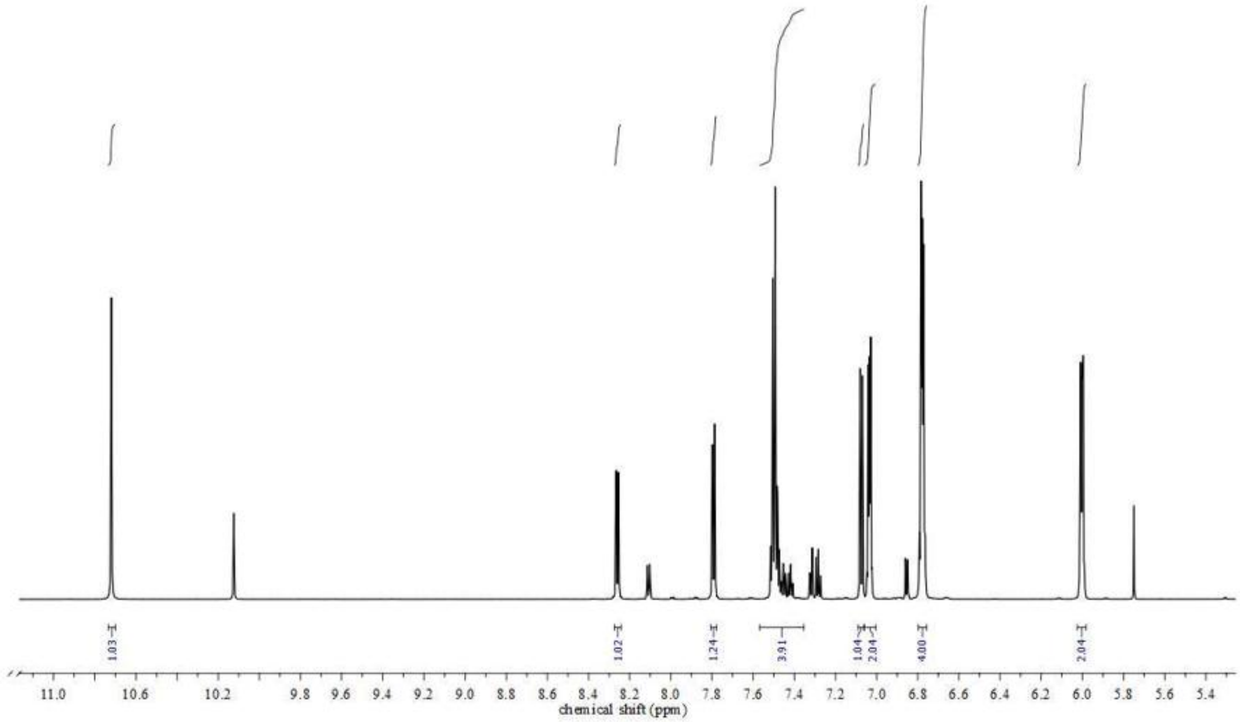

Figure S26. ^{1}H NMR (700 MHz, DMSO-d_6) of **5b**.

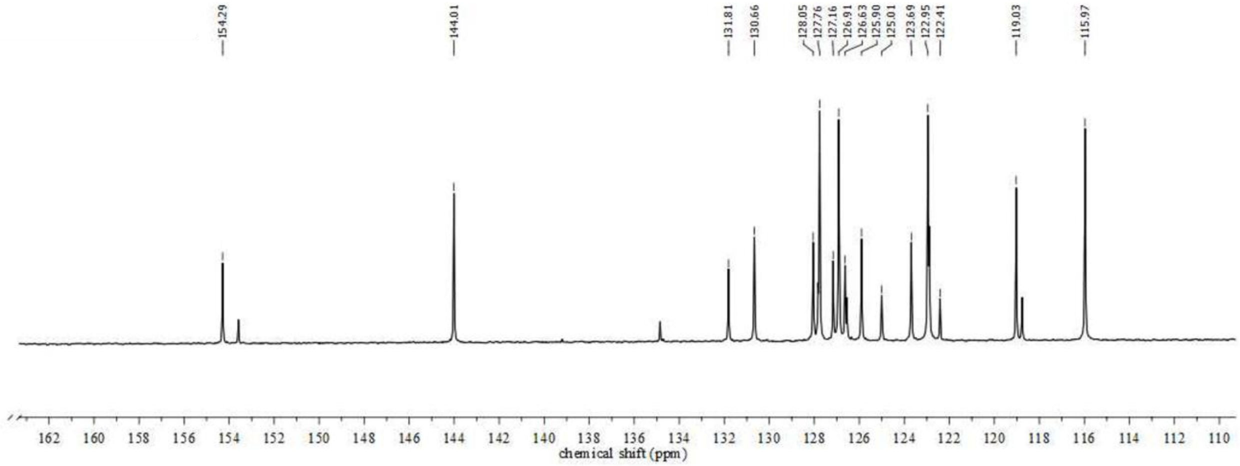

Figure S27. ^{13}C NMR (176 MHz, DMSO-d_6) of **5b**.

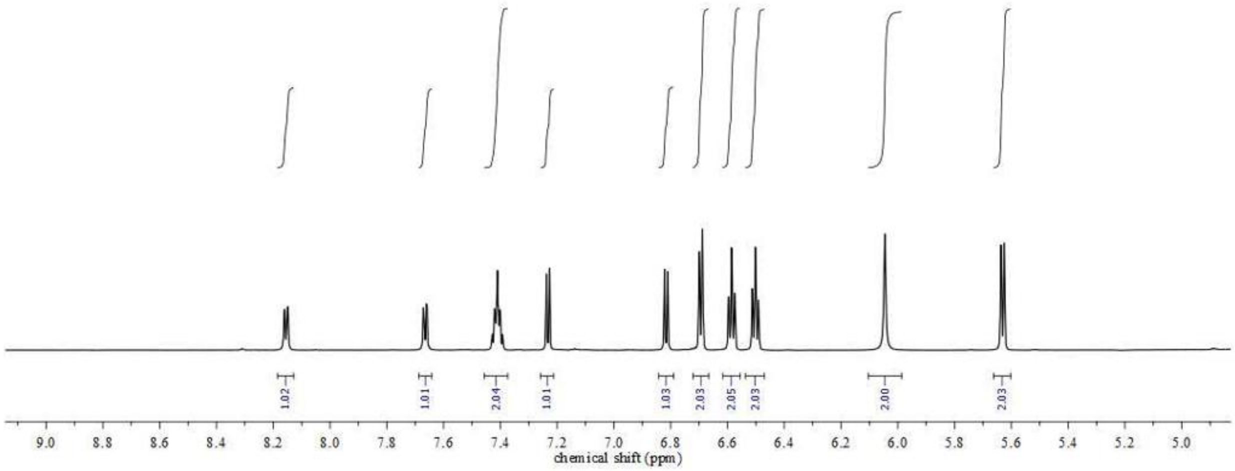

Figure S28. ^{1}H NMR (700 MHz, DMSO-d_6) of **6a**.

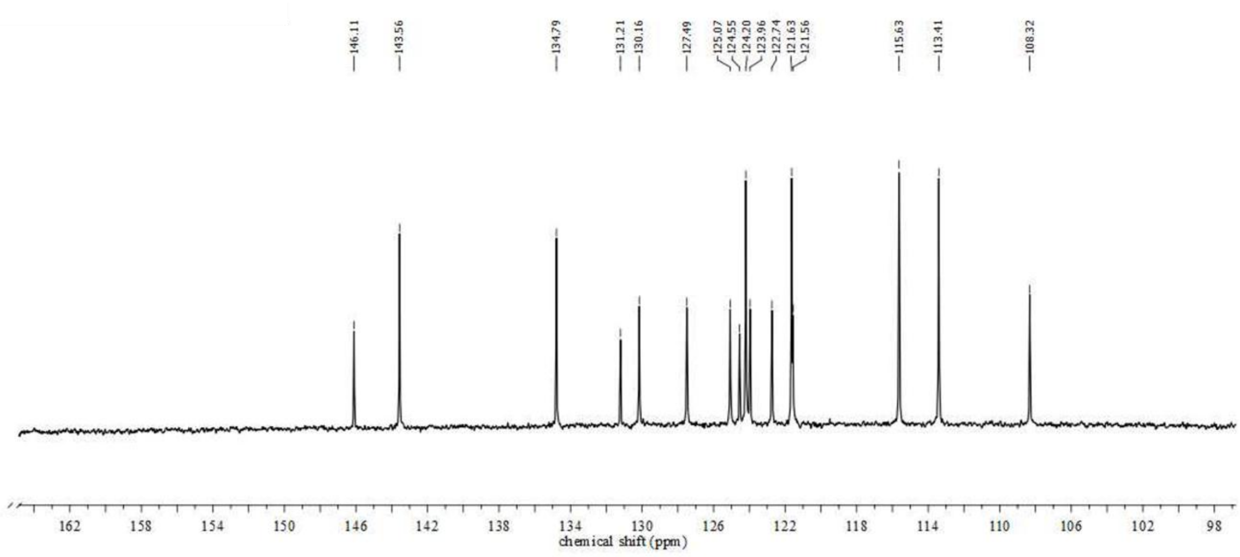

Figure S29. ^{13}C NMR (176 MHz, DMSO-d_6) of **6a**.

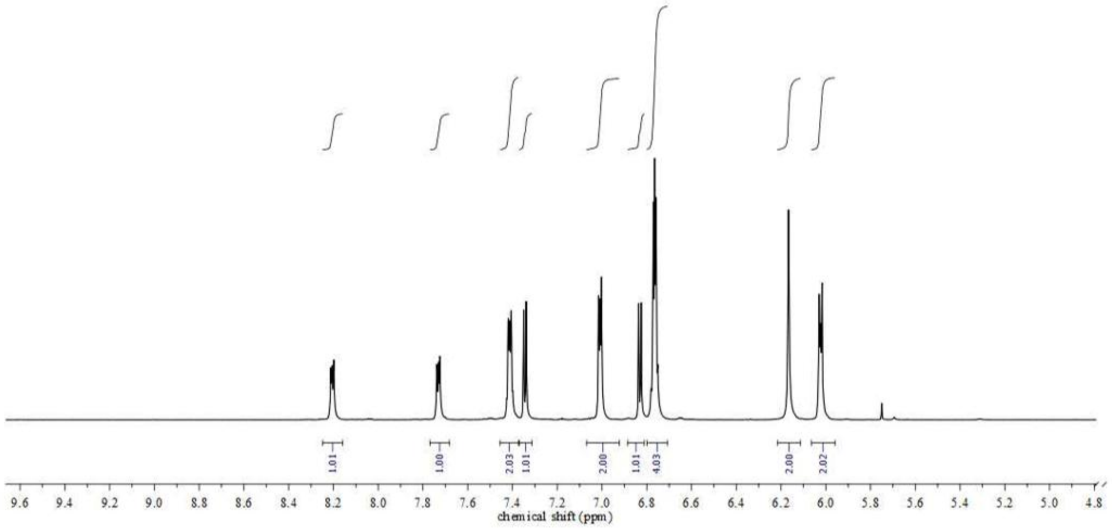

Figure S30. ^{1}H NMR (700 MHz, DMSO-d_6) of **6b**.

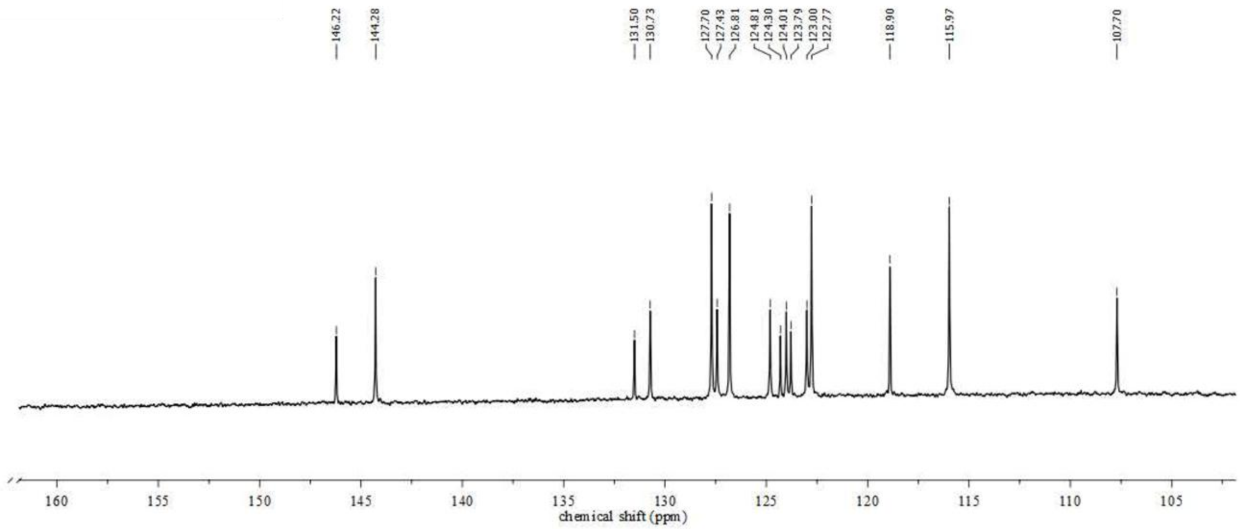

Figure S31. ^{13}C NMR (176 MHz, DMSO-d_6) of **6b**.

Computational Data

Mulliken charges and spin densities: POZ–H$^{\bullet}$

1	C	-0.098854	-0.066482
2	C	-0.184520	0.123976
3	C	-0.069451	-0.112607
4	C	0.030254	0.198959
5	C	-0.117981	-0.090401
6	C	-0.059774	0.182029
7	C	0.030585	0.198684
8	C	-0.069838	-0.112549
9	C	-0.184359	0.123991
10	H	0.134399	-0.008481
11	C	-0.098774	-0.066420
12	C	-0.059931	0.181962
13	C	-0.118961	-0.090305
14	H	0.130985	0.002900
15	H	0.134418	-0.008482
16	H	0.129438	0.003348
17	H	0.134293	-0.010675
18	H	0.130997	0.002900
19	H	0.134283	-0.010672
20	H	0.129449	0.003348
21	N	0.043642	0.465969
22	O	-0.100299	0.089008

Sum of Mulliken charges = 0.00000 1.00000

Mulliken charges and spin densities: $POZ^{\bullet+}$

1	C	-0.108342	0.024797
2	C	-0.400219	0.011457
3	C	-0.003845	0.064172
4	C	0.342872	0.138271
5	C	-0.142688	-0.073601
6	C	-0.003935	0.129351
7	C	0.343123	0.138042
8	C	-0.003114	0.064400
9	C	-0.401384	0.011354
10	H	0.149699	-0.002287
11	C	-0.108552	0.024858
12	C	-0.004014	0.129271
13	C	-0.143878	-0.073489
14	H	0.175181	-0.001856
15	H	0.149662	-0.002289
16	H	0.169556	0.002025
17	H	0.178530	-0.007195
18	H	0.175193	-0.001858
19	H	0.178520	-0.007192
20	H	0.169572	0.002024
21	N	0.005670	0.299677
22	O	-0.039627	0.145113
23	H	0.322022	-0.015042

Sum of Mulliken charges = 1.00000 1.00000

Mulliken charges and spin densities: PTZ–H$^{\bullet}$

1	C	-0.247621	-0.074559
2	C	-0.040506	0.126694
3	C	-0.180727	-0.088538
4	C	0.673418	0.202098
5	C	-0.409456	-0.115274
6	C	-0.058464	0.175875
7	C	0.674461	0.202113
8	C	-0.182380	-0.088677
9	C	-0.039372	0.126757
10	H	0.134153	-0.008000
11	C	-0.246736	-0.074531
12	C	-0.057956	0.175869
13	C	-0.409690	-0.115296
14	H	0.131150	0.003324
15	H	0.134208	-0.007999
16	H	0.128571	0.003568
17	H	0.131522	-0.010346
18	H	0.131165	0.003325
19	H	0.131519	-0.010347
20	H	0.128605	0.003571
21	N	0.115040	0.426818
22	S	-0.640902	0.143554

Sum of Mulliken charges = 0.00000 1.00000

Mulliken charges and spin densities: PTZ$^{\bullet+}$

1	C	-0.251312	0.022125
2	C	-0.253950	0.010105
3	C	-0.311405	0.100186
4	C	1.031300	0.132787
5	C	-0.419047	-0.096738
6	C	0.023925	0.099129
7	C	1.032118	0.132668
8	C	-0.311008	0.100244
9	C	-0.254150	0.010019
10	H	0.143295	-0.001680
11	C	-0.251173	0.022104
12	C	0.024212	0.099154
13	C	-0.418974	-0.096719
14	H	0.173674	-0.001642
15	H	0.143309	-0.001679
16	H	0.165875	0.001475
17	H	0.173201	-0.005728
18	H	0.173683	-0.001641
19	H	0.173196	-0.005728
20	H	0.165897	0.001476
21	N	0.125417	0.254447
22	H	0.301135	-0.012881
23	S	-0.379217	0.238516

Sum of charges = 1.00000 1.00000

Mulliken charges and spin densities: DMAC–H$^{\bullet}$

1	C	-0.230763	-0.092012
2	C	-0.110821	0.171608
3	C	-0.947714	-0.165063
4	C	0.576468	0.253256
5	C	0.143943	-0.103812
6	C	-0.279542	0.213720
7	C	0.579918	0.253301
8	C	-0.951501	-0.165226
9	C	-0.109663	0.171788
10	H	0.132183	-0.010051
11	C	-0.230755	-0.092010
12	C	-0.278982	0.213854
13	C	0.142249	-0.103924
14	H	0.127435	0.004529
15	H	0.132220	-0.010042
16	H	0.110244	0.004914
17	H	0.128080	-0.012398
18	H	0.127439	0.004531
19	H	0.128083	-0.012404
20	H	0.110197	0.004916
21	N	0.118645	0.501617
22	C	0.821154	-0.092489
23	C	-0.550518	0.026005
24	H	0.140663	0.001423
25	H	0.148696	0.001774
26	H	0.140715	0.001422
27	C	-0.548626	0.026004
28	H	0.141116	0.001471
29	H	0.148222	0.001812
30	H	0.141216	0.001487

Sum of Mulliken charges = 0.00000 1.00000

Mulliken charges and spin densities: DMAC$^{\bullet+}$

1	C	-0.208737	-0.059312
2	C	-0.324169	0.118695
3	C	-0.740559	-0.034661
4	C	0.550391	0.180094
5	C	0.132877	-0.083286
6	C	-0.214440	0.214645
7	C	0.553908	0.180172
8	C	-0.743251	-0.034642
9	C	-0.323632	0.118793
10	H	0.142374	-0.007130
11	C	-0.208931	-0.059340
12	C	-0.213973	0.214782
13	C	0.131341	-0.083383
14	H	0.168853	0.002585
15	H	0.142389	-0.007124
16	H	0.147490	0.003783
17	H	0.171235	-0.011640
18	H	0.168857	0.002588
19	H	0.171241	-0.011645
20	H	0.147439	0.003784
21	N	0.084441	0.349947
22	H	0.320559	-0.016504
23	C	0.917749	-0.021920
24	C	-0.486813	0.019080
25	H	0.166501	0.000631
26	H	0.165979	-0.000170
27	H	0.166526	0.000630
28	C	-0.485351	0.019406
29	H	0.166990	0.000660
30	H	0.165519	-0.000193
31	H	0.167199	0.000673

Sum of Mulliken charges = 1.00000 1.00000

POZ^+

Charge = 1 Multiplicity = 2

C	-4.07874	-1.71959	-0.17392
C	-2.68313	-1.74691	-0.05946
C	-1.95	-0.56035	0.07945
C	-2.64255	0.66062	0.07396
C	-4.02676	0.69021	-0.05038
C	-4.75343	-0.50392	-0.15937
C	-0.58807	1.86824	0.07801
C	0.142	0.66935	0.08357
C	1.53531	0.73277	-0.05117
H	2.10664	-0.19003	-0.01681
C	2.19006	1.96563	-0.16154
C	1.45591	3.14632	-0.14705
C	0.05916	3.09221	-0.03227
H	-4.62986	-2.65248	-0.26304
H	-2.15449	-2.69482	-0.02511
H	-4.52174	1.65388	-0.0446
H	-5.83608	-0.47453	-0.23282
H	3.27321	1.9936	-0.2475
H	1.9568	4.1066	-0.21738
H	-0.54225	3.99331	-0.03666
N	-0.55711	-0.53568	0.39366
O	-1.97303	1.87305	0.2524
H	-0.04725	-1.40311	-0.60269

Zero-point correction= 0.182236 (Hartree/Particle)
Thermal correction to Energy= 0.191901
Thermal correction to Enthalpy= 0.192845
Thermal correction to Gibbs Free Energy= 0.146565
Sum of electronic and zero-point Energies= -592.145018
Sum of electronic and thermal Energies= -592.135353
Sum of electronic and thermal Enthalpies= -592.134409
Sum of electronic and thermal Free Energies= -592.180689

POZ$^+$-O$_2^{\bullet-}$ (TS)

Charge = 0 Multiplicity = 1

C	-3.9828	-1.74962	-0.02428
C	-2.57779	-1.74289	-0.00456
C	-1.87559	-0.53055	-0.02153
C	-2.62552	0.66391	-0.03371
C	-4.00839	0.6589	-0.05185
C	-4.70329	-0.5606	-0.04812
C	-0.60218	1.9309	-0.04929
C	0.14011	0.72847	-0.04725
C	1.54169	0.82986	-0.08606
H	2.11838	-0.08053	-0.1063
C	2.16981	2.08021	-0.1113
C	1.41915	3.25147	-0.10486
C	0.01852	3.15898	-0.07292
H	-4.49762	-2.70805	-0.01323
H	-2.01082	-2.65858	0.04053
H	-4.52768	1.61177	-0.06238
H	-5.78719	-0.5682	-0.06123
H	3.25476	2.12994	-0.14695
H	1.89679	4.21946	-0.11466
H	-0.60008	4.05346	-0.06351
N	-0.4989	-0.49344	-0.00812
O	-1.99178	1.90481	-0.02204
H	0.2443	-1.6161	-0.17317
O	1.14302	-2.87891	-0.34438
O	0.57316	-3.7533	0.62941

Zero-point correction= 0.179239 (Hartree/Particle)
Thermal correction to Energy= 0.191379
Thermal correction to Enthalpy= 0.192324
Thermal correction to Gibbs Free Energy= 0.140748
Sum of electronic and zero-point Energies= -742.566046
Sum of electronic and thermal Energies= -742.553905
Sum of electronic and thermal Enthalpies= -742.552961
Sum of electronic and thermal Free Energies= -742.604537
IMAGINARY FREQUENCY: 1992.15 cm-1

POZ–H•

Charge = 0 Multiplicity = 2

C	-4.07874	-1.71959	-0.17392
C	-2.68313	-1.74691	-0.05946
C	-1.95	-0.56035	0.07945
C	-2.64255	0.66062	0.07396
C	-4.02676	0.69021	-0.05038
C	-4.75343	-0.50392	-0.15937
C	-0.58807	1.86824	0.07801
C	0.142	0.66935	0.08357
C	1.53531	0.73277	-0.05117
H	2.10664	-0.19003	-0.01681
C	2.19006	1.96563	-0.16154
C	1.45591	3.14632	-0.14705
C	0.05916	3.09221	-0.03227
H	-4.62986	-2.65248	-0.26304
H	-2.15449	-2.69482	-0.02511
H	-4.52174	1.65388	-0.0446
H	-5.83608	-0.47453	-0.23282
H	3.27321	1.9936	-0.2475
H	1.9568	4.1066	-0.21738
H	-0.54225	3.99331	-0.03666
N	-0.55711	-0.53568	0.39366
O	-1.97303	1.87305	0.2524

Zero-point correction= 0.168614 (Hartree/Particle)
Thermal correction to Energy= 0.178050
Thermal correction to Enthalpy= 0.178994
Thermal correction to Gibbs Free Energy= 0.133049
Sum of electronic and zero-point Energies= -591.783962
Sum of electronic and thermal Energies= -591.774526
Sum of electronic and thermal Enthalpies= -591.773582
Sum of electronic and thermal Free Energies= -591.819527

PTZ^{+}

Charge = 1 Multiplicity = 2

C	-4.00752	-1.85319	-0.10042
C	-2.63578	-1.72207	-0.11808
C	-2.04704	-0.44247	-0.08682
C	-2.87814	0.70374	-0.03679
C	-4.27309	0.5499	-0.01949
C	-4.83203	-0.71355	-0.05104
C	-0.51124	2.09506	-0.03144
C	0.08609	0.81143	-0.08252
C	1.49053	0.70363	-0.11012
H	1.94723	-0.27944	-0.14923
C	2.27218	1.83845	-0.08752
C	1.67735	3.11308	-0.0367
C	0.30152	3.23909	-0.00895
H	-4.45199	-2.84029	-0.12482
H	-1.99862	-2.59907	-0.15615
H	-4.90704	1.4281	0.01874
H	-5.90891	-0.82479	-0.03768
H	3.35092	1.74683	-0.1089
H	2.29831	3.99984	-0.01923
H	-0.15762	4.22002	0.03025
N	-0.67549	-0.33429	-0.10619
H	-0.16214	-1.2073	-0.14285
S	-2.24288	2.33161	0.00527

Zero-point correction= 0.179551 (Hartree/Particle)
Thermal correction to Energy= 0.189893
Thermal correction to Enthalpy= 0.190837
Thermal correction to Gibbs Free Energy= 0.142595
Sum of electronic and zero-point Energies= -915.123390
Sum of electronic and thermal Energies= -915.113048
Sum of electronic and thermal Enthalpies= -915.112104
Sum of electronic and thermal Free Energies= -915.160347

PTZ^{+}-O$_2^{\bullet-}$ (TS)

Charge = 0 Multiplicity = 1

C	-4.01752	-1.84319	-0.32042
C	-2.63578	-1.72207	-0.20808
C	-2.04704	-0.44247	-0.06682
C	-2.87814	0.70374	-0.03679
C	-4.27309	0.5499	-0.15949
C	-4.84203	-0.70355	-0.30104
C	-0.51124	2.09506	-0.03144
C	0.08609	0.81143	-0.06252
C	1.49053	0.70363	-0.21012
H	1.94723	-0.27944	-0.23923
C	2.27218	1.83845	-0.30752
C	1.67735	3.11308	-0.28671
C	0.30152	3.2391	-0.14895
H	-4.46199	-2.83029	-0.43482
H	-2.00862	-2.59907	-0.23615
H	-4.90704	1.4381	-0.13126
H	-5.91891	-0.81479	-0.39768
H	3.35092	1.74683	-0.4189
H	2.29831	4.00984	-0.36923
H	-0.15762	4.22002	-0.11975
N	-0.67549	-0.34429	0.05381
S	-2.23288	2.32161	0.36527
H	0.00835	-1.50725	-0.00503
O	0.80826	-2.84058	-0.08145
O	0.96986	-3.12057	1.3089

Zero-point correction= 0.176131 (Hartree/Particle)
Thermal correction to Energy= 0.188820
Thermal correction to Enthalpy= 0.189764
Thermal correction to Gibbs Free Energy= 0.136930
Sum of electronic and zero-point Energies= -1065.539230
Sum of electronic and thermal Energies= -1065.526541
Sum of electronic and thermal Enthalpies= -1065.525597
Sum of electronic and thermal Free Energies= -1065.578430
IMAGINARY FREQUENCY: 1932.24 cm-1

PTZ–H•

Charge = 0 Multiplicity = 2

C	-3.99113	-1.8411	-0.06745
C	-2.62139	-1.6839	-0.07644
C	-2.01484	-0.39788	-0.0573
C	-2.88863	0.7285	-0.02825
C	-4.27651	0.55871	-0.01926
C	-4.82874	-0.71443	-0.03865
C	-0.52775	2.11637	-0.02309
C	0.03155	0.80508	-0.0531
C	1.45019	0.70962	-0.06814
H	1.86865	-0.28927	-0.09108
C	2.25363	1.83004	-0.05432
C	1.67647	3.10985	-0.02452
C	0.29544	3.24651	-0.00911
H	-4.4222	-2.83544	-0.08267
H	-1.9521	-2.53535	-0.09857
H	-4.92217	1.43026	0.00298
H	-5.9058	-0.83282	-0.0315
H	3.33217	1.72326	-0.06635
H	2.30385	3.99326	-0.01343
H	-0.15208	4.23449	0.01384
N	-0.65826	-0.36346	-0.06892
S	-2.26848	2.37517	-0.00265

Zero-point correction= 0.165445 (Hartree/Particle)
Thermal correction to Energy= 0.175583
Thermal correction to Enthalpy= 0.176527
Thermal correction to Gibbs Free Energy= 0.128500
Sum of electronic and zero-point Energies= -914.754918
Sum of electronic and thermal Energies= -914.744780
Sum of electronic and thermal Enthalpies= -914.743836
Sum of electronic and thermal Free Energies= -914.791863

DMAC^{+}

Charge = 1 Multiplicity = 2

C	-4.02515	-1.75176	-0.4326
C	-2.65496	-1.66087	-0.33493
C	-2.05009	-0.39089	-0.19683
C	-2.80927	0.80446	-0.15418
C	-4.19243	0.66842	-0.25628
C	-4.79758	-0.58038	-0.393
C	-0.6279	2.0768	0.08439
C	0.04622	0.83173	0.03294
C	1.45394	0.73532	0.1157
H	1.92869	-0.23927	0.0719
C	2.205	1.88118	0.25046
C	1.55843	3.12599	0.30335
C	0.16904	3.21173	0.2212
H	-4.50187	-2.71788	-0.53905
H	-2.03477	-2.55061	-0.36302
H	-4.82173	1.54886	-0.22929
H	-5.87628	-0.64485	-0.46909
H	3.28405	1.82198	0.31466
H	2.14337	4.03159	0.40929
H	-0.29287	4.1897	0.26613
N	-0.68371	-0.32148	-0.10149
H	-0.1698	-1.19597	-0.13528
C	-2.14998	2.17373	-0.00222
C	-2.5278	3.05254	-1.23184
H	-2.09601	4.04975	-1.13688
H	-2.1656	2.60795	-2.16093
H	-3.61039	3.16473	-1.30409
C	-2.68364	2.84402	1.29901
H	-3.7685	2.95005	1.25731
H	-2.42991	2.25199	2.18062
H	-2.25602	3.84027	1.41926

Zero-point correction= 0.262188 (Hartree/Particle)
Thermal correction to Energy= 0.274934
Thermal correction to Enthalpy= 0.275878
Thermal correction to Gibbs Free Energy= 0.223468
Sum of electronic and zero-point Energies= -634.768891
Sum of electronic and thermal Energies= -634.756145
Sum of electronic and thermal Enthalpies= -634.755201
Sum of electronic and thermal Free Energies= -634.807611

DMAC^{+}-O$_2^{\bullet -}$ (TS)

Charge = 0 Multiplicity = 1

N	-0.26087	-0.59694	0.66483
C	0.65747	0.42544	0.54248
C	-0.94411	-0.68061	1.84844
C	1.57666	0.28938	-0.54048
C	0.75901	1.52192	1.41295
C	-0.99026	0.35004	2.82847
C	-1.63344	-1.91181	2.09336
C	2.51166	1.27696	-0.82052
H	1.52182	-0.61825	-1.13501
C	1.71186	2.5051	1.12287
C	-0.17773	1.62616	2.62347
C	-1.76759	0.1435	3.96217
H	-1.49262	-2.71337	1.37598
C	-2.41275	-2.06808	3.23723
C	2.57128	2.39472	0.01668
H	3.20661	1.15848	-1.63988
H	1.78808	3.38851	1.7556
C	0.67947	1.88809	3.90631
C	-1.15135	2.82966	2.41133
C	-2.50676	-1.02348	4.16076
H	-1.84048	0.93277	4.71381
H	-2.95715	-3.01089	3.4001
H	3.28236	3.17218	-0.18712
H	0.04029	2.0199	4.78088
H	1.37327	1.05108	4.08316
H	1.27561	2.8041	3.79839
H	-0.59643	3.77365	2.31805
H	-1.7443	2.69868	1.48396
H	-1.84806	2.92048	3.25676
H	-3.11371	-1.14528	5.05468
H	-0.42584	-1.79928	-0.21674
O	-0.59705	-3.04711	-1.13166
O	-1.26942	-2.45693	-2.24395

Zero-point correction= 0.258495 (Hartree/Particle)
Thermal correction to Energy= 0.273517
Thermal correction to Enthalpy= 0.274461
Thermal correction to Gibbs Free Energy= 0.217424
Sum of electronic and zero-point Energies= -785.159569
Sum of electronic and thermal Energies= -785.144548
Sum of electronic and thermal Enthalpies= -785.143604
Sum of electronic and thermal Free Energies= -785.200641
IMAGINARY FREQUENCY: 2271.38 cm-1

DMAC–H•

Charge = 0 Multiplicity = 2

C	-4.01253	-1.73854	-0.43163
C	-2.6394	-1.63721	-0.33182
C	-2.00507	-0.37521	-0.19194
C	-2.79986	0.80478	-0.15372
C	-4.18434	0.67027	-0.25678
C	-4.79325	-0.57676	-0.39398
C	-0.63277	2.06875	0.08378
C	0.01029	0.80013	0.02971
C	1.42543	0.73319	0.11572
H	1.87709	-0.25059	0.0708
C	2.18727	1.87654	0.25018
C	1.55332	3.12395	0.30293
C	0.16364	3.20561	0.22015
H	-4.4833	-2.70918	-0.53837
H	-2.00013	-2.51158	-0.35689
H	-4.81404	1.55271	-0.23008
H	-5.87268	-0.64199	-0.47081
H	3.26736	1.80992	0.31413
H	2.13939	4.02986	0.40842
H	-0.29968	4.185	0.26439
N	-0.64583	-0.38631	-0.10212
C	-2.154	2.1806	-0.00167
C	-2.53193	3.05933	-1.22669
H	-2.09775	4.05758	-1.13752
H	-2.16651	2.60958	-2.1525
H	-3.6156	3.17016	-1.30581
C	-2.68752	2.85075	1.29525
H	-3.77423	2.95459	1.25953
H	-2.43068	2.25334	2.17281
H	-2.25805	3.84687	1.42262

Zero-point correction= 0.248513 (Hartree/Particle)
Thermal correction to Energy= 0.260991
Thermal correction to Enthalpy= 0.261935
Thermal correction to Gibbs Free Energy= 0.209991
Sum of electronic and zero-point Energies= -634.398589
Sum of electronic and thermal Energies= -634.386111
Sum of electronic and thermal Enthalpies= -634.385166
Sum of electronic and thermal Free Energies= -634.437111

$O_2^{\bullet-}$

Charge = -1 Multiplicity = 2

O	-4.96197	-7.62771	-15.53866
O	-3.64197	-7.62771	-15.53866

Zero-point correction=	0.002953 (Hartree/Particle)
Thermal correction to Energy=	0.005325
Thermal correction to Enthalpy=	0.006269
Thermal correction to Gibbs Free Energy=	-0.016800
Sum of electronic and zero-point Energies=	-150.432745
Sum of electronic and thermal Energies=	-150.430373
Sum of electronic and thermal Enthalpies=	-150.429429
Sum of electronic and thermal Free Energies=	-150.452499

$HO_2^{\bullet}$

Charge = 0 Multiplicity = 2

O	-4.96197	-7.62771	-15.53866
O	-3.9007	-7.15546	-15.53866
H	-5.6223	-6.93088	-15.53866

Zero-point correction=	0.014571 (Hartree/Particle)
Thermal correction to Energy=	0.017421
Thermal correction to Enthalpy=	0.018365
Thermal correction to Gibbs Free Energy=	-0.007566
Sum of electronic and zero-point Energies=	-150.882671
Sum of electronic and thermal Energies=	-150.879820
Sum of electronic and thermal Enthalpies=	-150.878876
Sum of electronic and thermal Free Energies=	-150.904807

POZ-$PbBr_6$

Charge = -3 Multiplicity = 2

C	-4.22807	-0.42321	1.47033
C	-3.00311	-0.89248	1.00448
C	-1.81539	-0.28337	1.42016
C	-1.89741	0.8311	2.27492
C	-3.11556	1.30645	2.71977
C	-4.29799	0.67051	2.32793
C	0.35703	1.31834	1.87671
C	0.46236	0.20854	1.03378
C	1.57112	0.08962	0.20086
H	1.65385	-0.75653	-0.47221
C	2.56778	1.05805	0.24069
C	2.46381	2.15349	1.09426
C	1.34032	2.28888	1.91473
H	-5.13775	-0.91298	1.13952
H	-2.93134	-1.71694	0.3024
H	-3.1199	2.16888	3.37761
H	-5.25477	1.03675	2.68355
H	3.41218	0.93123	-0.42778
H	3.24242	2.90863	1.12158
H	1.21351	3.13179	2.5856
N	-0.57201	-0.7229	1.03391
O	-0.74233	1.43982	2.72667
H	-0.52872	-1.43193	0.29466
Pb	2.17446	-4.46365	-0.44001
Br	-0.42192	-3.18127	-1.52925
Br	0.57364	-6.56187	0.76171
Br	2.50512	-6.00732	-2.87218
Br	4.57773	-5.51467	0.76335
Br	1.61303	-2.69564	1.88974
Br	3.7574	-2.21153	-1.65199

Zero-point correction= 0.184346 (Hartree/Particle)
Thermal correction to Energy= 0.210653
Thermal correction to Enthalpy= 0.211597
Thermal correction to Gibbs Free Energy= 0.114967
Sum of electronic and zero-point Energies= -674.846378
Sum of electronic and thermal Energies= -674.820071
Sum of electronic and thermal Enthalpies= -674.819127
Sum of electronic and thermal Free Energies= -674.915756

POZ-PbBr$_6$ (TS)

Charge = -3 Multiplicity = 2

H	-0.41216	-1.57791	0.08698
Pb	2.12771	-4.51993	-0.47339
Br	-0.40192	-3.47127	-1.62925
Br	0.74143	-6.80029	0.69065
Br	2.61726	-5.97334	-2.93588
Br	4.63405	-5.41749	0.73683
Br	1.40694	-2.83251	1.92697
Br	3.61587	-2.0957	-1.55767
N	-0.48412	-0.65086	1.00057
C	0.21402	0.5854	0.84855
C	-1.25537	-0.836	2.19722
C	1.27012	0.75775	-0.05583
C	-0.32361	1.71358	1.49803
C	-1.73789	0.30855	2.81285
C	-1.69169	-2.08925	2.63343
C	1.77206	2.03991	-0.31922
H	1.66843	-0.10439	-0.57377
C	0.18062	2.98536	1.2356
O	-1.40495	1.59246	2.36864
C	-2.64	0.20234	3.87393
H	-1.35189	-2.98143	2.11265
C	-2.59151	-2.19909	3.70244
C	1.21771	3.16098	0.31699
H	2.58009	2.17276	-1.03033
H	-0.2668	3.8251	1.74472
C	-3.07612	-1.0562	4.32082
H	-2.98899	1.11625	4.34227
H	-2.92678	-3.18561	4.01947
H	1.59314	4.14752	0.09887
H	-3.77999	-1.12341	5.13848

Zero-point correction= 0.176810 (Hartree/Particle)
Thermal correction to Energy= 0.202580
Thermal correction to Enthalpy= 0.203524
Thermal correction to Gibbs Free Energy= 0.108995
Sum of electronic and zero-point Energies= -674.793658
Sum of electronic and thermal Energies= -674.767888
Sum of electronic and thermal Enthalpies= -674.766944
Sum of electronic and thermal Free Energies= -674.861473
IMAGINARY FREQUENCY: 1774.40 cm-1

PTZ-$PbBr_6$

Charge = -3 Multiplicity = 2

Pb	2.09074	-4.64663	-0.44702
Br	-0.44192	-3.44127	-1.51925
Br	1.2191	-7.27878	0.53902
Br	2.62864	-5.81942	-3.0402
Br	4.77998	-5.16091	0.6296
Br	0.82121	-3.46012	2.08436
Br	3.27417	-1.97047	-1.24346
H	-0.13058	-1.67091	-0.2086
N	-0.12579	-0.67773	0.65447
C	-0.84248	-0.83573	1.83074
C	0.58569	0.46282	0.24674
C	-1.7186	-1.95112	1.9419
C	-0.80365	0.11338	2.88092
C	0.77495	1.5639	1.11015
C	1.01772	0.55515	-1.10622
C	-2.46255	-2.14	3.09179
H	-1.82571	-2.63256	1.11268
C	-1.52501	-0.13898	4.06494
S	-0.41851	1.88532	2.51242
C	1.48024	2.70851	0.64726
H	0.81431	-0.25352	-1.7868
C	1.64547	1.71793	-1.56452
C	-2.37265	-1.21614	4.16432
H	-3.13873	-2.99962	3.16533
H	-1.48589	0.60385	4.87177
C	1.87349	2.80095	-0.67565
H	1.60198	3.57489	1.30944
H	1.91625	1.80994	-2.61922
H	-2.93993	-1.3848	5.08257
H	2.34576	3.69722	-1.05013

Zero-point correction= 0.181806 (Hartree/Particle)
Thermal correction to Energy= 0.208574
Thermal correction to Enthalpy= 0.209518
Thermal correction to Gibbs Free Energy= 0.112776
Sum of electronic and zero-point Energies= -997.830067
Sum of electronic and thermal Energies= -997.803299
Sum of electronic and thermal Enthalpies= -997.802354
Sum of electronic and thermal Free Energies= -997.899096

PTZ-$PbBr_6$ (TS)

Charge = -3 Multiplicity = 2

Pb	2.09074	-4.64663	-0.44702
Br	-0.44192	-3.44127	-1.51925
Br	1.2191	-7.27878	0.53902
Br	2.62864	-5.81942	-3.0402
Br	4.77998	-5.16091	0.6296
Br	0.82121	-3.46012	2.08436
Br	3.27417	-1.97047	-1.24346
H	-0.13058	-1.67091	-0.2086
N	-0.12579	-0.67773	0.65447
C	-0.84248	-0.83573	1.83074
C	0.58569	0.46282	0.24674
C	-1.7186	-1.95112	1.9419
C	-0.80365	0.11338	2.88092
C	0.77495	1.5639	1.11015
C	1.01772	0.55515	-1.10622
C	-2.46255	-2.14	3.09179
H	-1.82571	-2.63256	1.11268
C	-1.52501	-0.13898	4.06494
S	-0.41851	1.88532	2.51242
C	1.48024	2.70851	0.64726
H	0.81431	-0.25352	-1.7868
C	1.64547	1.71793	-1.56452
C	-2.37265	-1.21614	4.16432
H	-3.13873	-2.99962	3.16533
H	-1.48589	0.60385	4.87177
C	1.87349	2.80095	-0.67565
H	1.60198	3.57489	1.30944
H	1.91625	1.80994	-2.61922
H	-2.93993	-1.3848	5.08257
H	2.34576	3.69722	-1.05013

Zero-point correction= 0.172487 (Hartree/Particle)
Thermal correction to Energy= 0.198959
Thermal correction to Enthalpy= 0.199903
Thermal correction to Gibbs Free Energy= 0.104952
Sum of electronic and zero-point Energies= -997.754425
Sum of electronic and thermal Energies= -997.727953
Sum of electronic and thermal Enthalpies= -997.727009
Sum of electronic and thermal Free Energies= -997.821961
IMAGINARY FREQUENCY: 846.01 cm-1

DMAC-PbBr$_6$

Charge = -3 Multiplicity = 2

Pb	2.2616	-4.68971	-0.92788
Br	-0.4203	-3.20005	-1.07407
Br	0.90853	-6.83342	0.49463
Br	1.75685	-5.97088	-3.4819
Br	4.83201	-5.97187	-0.60751
Br	2.57461	-3.18395	1.61957
Br	3.52783	-2.3691	-2.34808
H	0.26153	-1.64723	0.70086
N	0.13825	-0.77057	1.21601
C	0.96924	0.27922	0.91559
C	-0.81198	-0.71381	2.18502
C	1.96276	0.0851	-0.05962
C	0.83276	1.51475	1.56673
C	-1.04552	0.47282	2.90768
C	-1.56706	-1.87319	2.46283
C	2.80651	1.12309	-0.39669
H	2.06979	-0.88586	-0.53504
C	1.70414	2.54562	1.20071
C	-0.22338	1.74041	2.65296
C	-2.05088	0.46354	3.8764
H	-1.34325	-2.77148	1.89441
C	-2.55167	-1.84194	3.42844
C	2.68248	2.36815	0.23015
H	3.56748	0.93911	-1.1478
H	1.61739	3.51215	1.69105
C	0.48192	2.15309	3.9631
C	-1.16949	2.87904	2.21487
C	-2.81025	-0.66895	4.14696
H	-2.24637	1.37202	4.44036
H	-3.12042	-2.74341	3.63153
H	3.34626	3.18705	-0.02801
H	-0.24687	2.32941	4.76042
H	1.1602	1.3591	4.28141
H	1.06366	3.06823	3.8188
H	-0.60888	3.80192	2.03873
H	-1.67836	2.60249	1.28916
H	-1.92579	3.07382	2.98194
H	-3.58284	-0.64171	4.9079

Zero-point correction= 0.264524 (Hartree/Particle)
Thermal correction to Energy= 0.294111
Thermal correction to Enthalpy= 0.295055
Thermal correction to Gibbs Free Energy= 0.190854
Sum of electronic and zero-point Energies= -717.471360
Sum of electronic and thermal Energies= -717.441774
Sum of electronic and thermal Enthalpies= -717.440829
Sum of electronic and thermal Free Energies= -717.545030

DMAC-PBBr$_6$ (TS)

Charge = -3 Multiplicity = 2

Pb	2.13417	-4.5638	-0.4603
Br	-0.49158	-3.44166	-1.44881
Br	0.98732	-7.01707	0.607
Br	2.53977	-5.83589	-3.03427
Br	4.76564	-5.35467	0.64239
Br	1.19684	-3.07893	2.00295
Br	3.66304	-2.02613	-1.50844
H	-0.42058	-1.76091	-0.1886
N	-0.26087	-0.59694	0.66483
C	0.65747	0.42544	0.54248
C	-0.94411	-0.68061	1.84844
C	1.57666	0.28938	-0.54048
C	0.75901	1.52192	1.41295
C	-0.99026	0.35004	2.82847
C	-1.63344	-1.91181	2.09336
C	2.51166	1.27696	-0.82052
H	1.52182	-0.61825	-1.13501
C	1.71186	2.5051	1.12287
C	-0.17773	1.62616	2.62347
C	-1.76759	0.1435	3.96217
H	-1.49262	-2.71337	1.37598
C	-2.41275	-2.06808	3.23723
C	2.57128	2.39472	0.01668
H	3.20661	1.15848	-1.63988
H	1.78808	3.38851	1.7556
C	0.67947	1.88809	3.90631
C	-1.15135	2.82966	2.41133
C	-2.50676	-1.02348	4.16076
H	-1.84048	0.93277	4.7138
H	-2.95715	-3.01089	3.4001
H	3.28236	3.17218	-0.18712
H	0.04029	2.0199	4.78088
H	1.37327	1.05108	4.08315
H	1.27561	2.8041	3.79839
H	-0.59643	3.77365	2.31805
H	-1.7443	2.69868	1.48396
H	-1.84806	2.92048	3.25676
H	-3.11371	-1.14528	5.05468

Zero-point correction= 0.256143 (Hartree/Particle)
Thermal correction to Energy= 0.284965
Thermal correction to Enthalpy= 0.285909
Thermal correction to Gibbs Free Energy= 0.185015
Sum of electronic and zero-point Energies= -717.404927
Sum of electronic and thermal Energies= -717.376104
Sum of electronic and thermal Enthalpies= -717.375160
Sum of electronic and thermal Free Energies= -717.476054
IMAGINARY FREQUENCY: 1372.35 cm-1

$PbBr_4^{2-}$

Charge = -2 Multiplicity = 1

Pb	2.09074	-4.64663	-0.44702
Br	1.2191	-7.27878	0.53902
Br	2.62864	-5.81942	-3.0402
Br	4.77998	-5.16091	0.6296
Br	3.27417	-1.97047	-1.24346

Zero-point correction= 0.001428 (Hartree/Particle)
Thermal correction to Energy= 0.011455
Thermal correction to Enthalpy= 0.012399
Thermal correction to Gibbs Free Energy= -0.045955
Sum of electronic and zero-point Energies= -56.218938
Sum of electronic and thermal Energies= -56.208911
Sum of electronic and thermal Enthalpies= -56.207967
Sum of electronic and thermal Free Energies= -56.266321

HBr

Charge = 0 Multiplicity = 1

Br	4.0028	-2.30759	-0.51625
H	5.08135	-1.35552	-0.57869

Zero-point correction= 0.005919 (Hartree/Particle)
Thermal correction to Energy= 0.008279
Thermal correction to Enthalpy= 0.009223
Thermal correction to Gibbs Free Energy= -0.013319
Sum of electronic and zero-point Energies= -13.705737
Sum of electronic and thermal Energies= -13.703376
Sum of electronic and thermal Enthalpies= -13.702432
Sum of electronic and thermal Free Energies= -13.724975

Br-

Charge = -1 Multiplicity = 1

Br	3.41789	8.07248	-1.96402

Zero-point correction= 0.000000 (Hartree/Particle)
Thermal correction to Energy= 0.001416
Thermal correction to Enthalpy= 0.002360
Thermal correction to Gibbs Free Energy= -0.016176
Sum of electronic and zero-point Energies= -13.186382
Sum of electronic and thermal Energies= -13.184966
Sum of electronic and thermal Enthalpies= -13.184021
Sum of electronic and thermal Free Energies= -13.202557
